# Genome reorganization during emergence of host-associated *Mycobacterium abscessus*

**DOI:** 10.1099/mgen.0.000706

**Published:** 2021-12-07

**Authors:** Lindsey L. Bohr, Madison A. Youngblom, Vegard Eldholm, Caitlin S. Pepperell

**Affiliations:** ^1^​ Department of Medical Microbiology and Immunology, School of Medicine and Public Health, University of Wisconsin-Madison, Madison, WI, USA; ^2^​ Norwegian Institute of Public Health, Oslo, Norway; ^3^​ Department of Medicine, School of Medicine and Public Health, University of Wisconsin-Madison, Madison, WI, USA

**Keywords:** evolution, lateral gene transfer, *Mycobacterium abscessus*, NTM infection, recombination

## Abstract

*
Mycobacterium abscessus* is a rapid growing, free-living species of bacterium that also causes lung infections in humans. Human infections are usually acquired from the environment; however, dominant circulating clones (DCCs) have emerged recently in both *M. abscessus* subsp. *massiliense* and subsp. *abscessus* that appear to be transmitted among humans and are now globally distributed. These recently emerged clones are potentially informative about the ecological and evolutionary mechanisms of pathogen emergence and host adaptation. The geographical distribution of DCCs has been reported, but the genomic processes underlying their transition from environmental bacterium to human pathogen are not well characterized. To address this knowledge gap, we delineated the structure of *M. abscessus* subspecies *abscessus* and *massiliense* using genomic data from 200 clinical isolates of *M. abscessus* from seven geographical regions. We identified differences in overall patterns of lateral gene transfer (LGT) and barriers to LGT between subspecies and between environmental and host-adapted bacteria. We further characterized genome reorganization that accompanied bacterial host adaptation, inferring selection pressures acting at both genic and intergenic loci. We found that both subspecies encode an expansive pangenome with many genes at rare frequencies. Recombination appears more frequent in *M. abscessus* subsp. *massiliense* than in subsp. *abscessus*, consistent with prior reports. We found evidence suggesting that phage are exchanged between subspecies, despite genetic barriers evident elsewhere throughout the genome. Patterns of LGT differed according to niche, with less LGT observed among host-adapted DCCs versus environmental bacteria. We also found evidence suggesting that DCCs are under distinct selection pressures at both genic and intergenic sites. Our results indicate that host adaptation of *M. abscessus* was accompanied by major changes in genome evolution, including shifts in the apparent frequency of LGT and impacts of selection. Differences were evident among the DCCs as well, which varied in the degree of gene content remodelling, suggesting they were placed differently along the evolutionary trajectory toward host adaptation. These results provide insight into the evolutionary forces that reshape bacterial genomes as they emerge into the pathogenic niche.

## Data Summary

Previously published sequencing data used in this study are available from the National Center for Biotechnology Information (NCBI) under the Sequence Read Archive (SRA) accession numbers found in Table S1 (available with the online version of this article).

**Impact Statement**

*
Mycobacterium abscessus* is an important pathogen that causes skin abscesses, and pulmonary and other infections, and is a common bacterial pathogen isolated from cystic fibrosis patients. Until recently, *M. abscessus* was thought to be acquired exclusively from the environment; however, new evidence of person-to-person transmission has serious implications for how we monitor and treat these infections, particularly with antibiotic-resistant strains. Our study leverages powerful genomics tools to characterize the recent emergence of *M. abscessus* into the human pathogenic niche. We find significant genomic changes associated with strains originating from person-to-person transmission including changes in patterns of lateral gene transfer, and evidence for relaxed purifying selection. Our work highlights the importance of genome remodelling in the process of pathogen emergence.

## Introduction

*Mycobacterium abscessus* is a rapid growing mycobacterial species found in a variety of environments [1, 2]. This free-living bacterium is an opportunistic pathogen capable of causing skin abscesses, and pulmonary and other infections [1, 3]. *M. abscessus* is the most common species of non-tuberculous mycobacterium (NTM) isolated from individuals with cystic fibrosis (CF) [4]. *M. abscessus* infections are hard to treat, as the bacteria are intrinsically resistant to many antibiotics and additional resistances are on the rise [5–7]. Until recently, infection with *M. abscessus* was thought to be exclusively acquired from the environment; however, evidence of person-to-person transmission has been identified for recently emerged dominant circulating clones (DCCs) [4, 8, 9]. DCCs are genetically homogeneous groups of bacteria that are globally distributed, and account for a substantial proportion of infections in CF patients; the circumstances in which these organisms may be transmitted person to person are as yet unclear [10, 11].

*M. abscessus* is distantly related to *Mycobacterium tuberculosis* and other pathogenic mycobacteria. *M. abscessus* has been divided into three subspecies: *abscessus*, *massiliense* and *bolletii* [12]. Average nucleotide identity (ANI) values within and between subspecies of *M. abscessus* are consistent as described in other species: within subspecies ANI values are >98 %, while between subspecies ANI values are ~96–97 % [12, 13]. It was recently reported that *M. abscessus* subsp. *abscessus* recombines more frequently than the other subspecies [13]. *M. abscessus* encodes a diverse array of plasmids and prophages [8, 14–16]; the extent to which distinct mechanisms of lateral gene transfer (LGT) shape diversity of *M. abscessus* is unknown.

The recent emergence of host-adapted lineages of *M. abscessus* provides an opportunity to learn about the mechanisms underlying bacterial pathogen emergence, including the role of LGT. LGT can enable bacterial adaptation to new environments, including the pathogenic niche. Emergence of the major pathogen *M. tuberculosis* was accompanied by acquisition of novel genetic material by LGT [17–19], followed by apparent loss of the capacity to engage in LGT [20–23]. Previous research points to a reduction in genome size, an increase in pseudogenes and a loss of metabolic capacity in association with bacterial pathogen emergence [24–27]. Our aim here was to use comparative genomics to investigate genome reorganization occurring during emergence of host-associated *M. abscessus *. We found putatively host-adapted DCCs to be characterized by lower levels of LGT relative to environmentally acquired *M. abscessus *. We did not observe large-scale pseudogenization across the DCCs; however, we found evidence of relaxation of purifying selection in the core and accessory genomes of DCCs, which may be an earlier indicator of host adaptation. Taken together, these results provide insight into genome reorganization that accompanies bacterial pathogen emergence.

## Methods

### Data set and subsampling

We compiled Illumina sequence read data from a collection of 554 clinical isolates including newly sequenced isolates from Norway (PRJEB9515), as well as publicly available isolates from Michigan (PRJNA315990), Maryland (PRJNA231221), and the sample published by Bryant *et al*. [8]. We mapped raw sequence reads to the *M. abscessus* reference ATCC 19977 genome. Our pipeline can be found at https://github.com/pepperell-lab/RGAPepPipe (accessed 20 July 2021). Briefly, we used FastQC v0.11.8 [28] and TrimGalore v0.6.4 [29] for quality assessment and read adaptor trimming. We mapped the trimmed reads to the *M. abscessus* ATCC 19977 genome using SAMtools v1.5 [30], and used Picard Tools v1.183 for sorting and format conversion (http://broadinstitute.github.io/picard/). Lastly, we called variants using Pilon v1.16 [31]. Once all isolates were aligned to the reference, we separated the *M. abscessus* isolates by subsp. *abscessus* and *massiliense*, and used FastTree v2.1.9 [32] to reconstruct a maximum-likelihood phylogeny under the discrete gamma distribution model. Then, we used TreeGubbins v2.4.1 [33] to identify the DCCs: *M. abscessus* subsp. *abscessus* DCC1 and DCC2, and *M. abscessus* subsp. *massiliense* DCC1, following the nomenclature described by Bryant *et al*. [8]. We labelled the non-DCC isolates as environmentally acquired isolates (EAIs). We used Treemer v0.3 [34] to informatively subsample based on maintaining maximum phylogenetic diversity, and selected 40 DCCs and 40 EAIs for each group; this resulted in a final dataset of 120 *M. abscessus* subsp. *abscessus* and 80 subsp. *massiliense* isolates. We included isolates from subspecies *abscessus* and *massiliense*. We did not include isolates from *M. abscessus* subspecies *bolletii *, because there were few isolates available and there have not been reports of host-associated DCC emergences within the subspecies [8]. The final data set spans seven geographical regions: Australia, Denmark, Ireland, Holland, Norway, the UK and the USA. Isolate metadata and accession numbers can be found in Table S1.

### DNA extraction and sequencing

Bacterial isolates received at the Norwegian Institute of Public Health were grown on Lowenstein–Jensen media. Genomic DNA was extracted as described in Eldholm *et al*. [35]. Sequencing libraries were prepared using the Nextera XT kit (Illumina) as per the manufacturer’s protocol and sequenced on the Illumina MiSeq platform generating paired-end 250 bp reads.

### *De novo* assembly, annotation, quality control and core-genome alignment

The *M. abscessus* genomes were *de novo* assembled with SPAdes v. 3.12.0 [36] and annotated using Prokka v 1.14-dev [37]. We assessed the quality of the *de novo* assemblies using Quast v. 4.6.3 [38] and removed isolates with N50 <50 kbp. We identified orthologous genes in the core and accessory genomes using Roary v. 3.11.2 [39] with a amino acid identity of 95%, and we aligned the core genes with prank [40]. Additionally, for downstream intergenic region (IGR) analyses, we re-analysed the core genes using Roary without splitting paralogous genes.

### Maximum-likelihood phylogenetic analysis

We performed maximum-likelihood phylogenetic inference on core-genome alignments using RAxML v 8.2.3 [41] with the GTR (general time reversible) model of nucleotide substitution and gamma distribution of rate variation. We performed bootstrapping using autoMR convergence. We used ggtree [42] for tree visualization.

### Recombination detection in the core genome

Recombinant tracts were identified in the core genomes using Gubbins v 2.3.2 [43] and visualized with Phandango [44]. Briefly, Gubbins identifies recombination by using spatial scanning statistics to identify loci with high densities of SNPs. We calculated the proportion of sites affected by recombination per isolate and performed a Kruskal–Wallis test in R to determine the differences in proportion of recombinant sites by group [45]. We then performed pairwise Mann–Whitney–Wilcoxon tests in R with Bonferroni correction to identify which distribution pairs were significantly different [46, 47]. Additionally, we compared the fragment length distributions of recombinant tracts between *M. abscessus* subspecies to inferred recombinant tracts in other mycobacterial species known to participate in distributive conjugal transfer (DCT): '*Mycobacterium canettii'* [48, 49] and *Mycobacterium smegmatis* [50, 51]. We used SplitsTree4 v. 4.14.4 [52] to reconstruct a phylogenetic network of the core-genome alignments for both subspecies and conducted a PHI (pairwise homoplasy index) test to detect recombination [53].

### Admixture analyses

We performed bacterial admixture analyses using FineSTRUCTURE v. 2.1.3 [54] to investigate the extent of LGT among *M. abscessus* subspecies. We constructed a core biallelic SNP alignment across all 200 *M *. *abscessus* isolates using SNP-sites [55] and VCFtools [56]. Next, we used FineSTRUCTURE to build a co-ancestry matrix that contains the number of recombination events from each donor to each recipient. Briefly, FineSTRUCTURE infers fragments transferred via recombination and reconstructs haplotypes on the chromosome of a recipient as a series of fragments from all other donor individuals within the sample [54]. Additionally, we performed these analyses using core biallelic SNP alignments of each subspecies separately to compare recombination events within and between the DCCs and EAIs.

### Pangenome selection and diversity analyses

We calculated pangenome accumulation and rarefaction curves of *M. abscessus* subsp. *abscessus* and subsp. *massiliense* isolates, as well as DCCs and EAIs for both subspecies using random subsampling without replacement 100 times, plotting the median value for the core and pangenome values. Additionally, we calculated the gene frequencies of accessory genes from both subspecies. Using EggLib [57], we calculated the mean π per accessory gene within and between subspecies using a subset of genes found at intermediate frequencies (1–99 %) in both species. We performed a Kruskal–Wallis test in R to determine the differences in mean gene π values by group [45]. We then performed pairwise Mann–Whitney–Wilcoxon tests in R with Bonferroni correction to identify significant differences among distribution pairs [46, 47]. To investigate the effects of LGT and selection on diversity within subspecies, we calculated the distribution of pairwise πN/πS values across the accessory genes present in at least four isolates per group [57].

### Core-genome and accessory-genome distances

Core- and accessory-genome tree distances were calculated for all pairs of isolates within a subspecies using the distTips function from the adephylo (https://cran.r-project.org/web/packages/adephylo/index.html) package in R. For core distances, the separate core-genome phylogenies for each subspecies were used (see the *Maximum-likelihood phylogenetic analysis* section in Methods). For accessory distances, we used the phylogenetic tree based on accessory gene presence/absence as output by Roary (see the *De novo assembly, annotation, quality control and core-genome alignment* section in Methods).

In order to measure core-genome divergence for samples, as opposed to pairs of isolates, we calculated segregating sites for the separate core-genome alignments of *M. abscessus* subsp. *abscessus* and subsp. *massiliense*. The number of segregating sites (normalized to the overall size of the core-genome alignment) was measured for random subsamples of EAIs and DCC isolates (100 repetitions for each isolate type, *N*=10 sampled without replacement). From each subsample, the core-genome and pangenome sizes (as calculated by Roary) were plotted against the number of segregating sites calculated using EggLib [57]. A linear regression with a 95 % confidence interval was fit to the data from the EAIs using the stat_smooth() function in ggplot2 with the lm method. Regression equations, *R* ^2^ values and *P* values were plotted using the stat_poly_eq() and stat_fit_glance() methods from ggpmisc (https://cran.r-project.org/web/packages/ggpmisc/index.html).

### Prophage identification

We used ProphET [58] to detect prophage in our collection of genomes. Briefly, ProphET performs a similarity search of annotated proteins from bacterial genomes against a database of known phage proteins to identify prophage within bacterial genomes. ProphET discards regions with a low density of phage-associated genes. After identifying the prophage regions, we calculated pairwise mash [59] distances, which are based on shared *k*-mer (sequences of length *k*) content between prophage nucleotide sequences. To visualize the relatedness between prophage regions, we plotted a dendrogram in R.

### Pseudogene analysis

To identify and characterize possible pseudogenes, we looked for identity between intergenic sequences in our alignment and known protein sequences. Specifically, we constructed an alignment of all IGRs using Piggy [60] with a minimum per cent length identity and minimum per cent nucleotide identity of 90 %. Next, we created a custom protein blast [61] database of all publicly available *M. abscessus* proteins and blasted the IGRs against the custom database. Pseudogenes were identified if the blast result had an *E* value cut-off of 10e^−5^ as described by Belda and colleagues [62]. We performed a Kruskal–Wallace test in R to determine differences in mean pseudogene values by group [45]. We then performed pairwise Mann–Whitney–Wilcoxon tests in R with Bonferroni correction to identify significant differences among distribution pairs [47, 49].

### IGR selection and diversity analyses

To identify the extent to which selection is acting on IGRs in the DCCs of both *M. abscessus* subsp. *abscessus* and subsp. *massiliense*, we estimated dI/dS across intergenic alignments of *M. abscessus* subsp. *abscessus* DCC1, DCC2 and EAI, and *M. abscessus* subsp. *massiliense* DCC and EAIs, as described previously [63]. Briefly, we calculated dN/dS across the core-gene alignments using the yn00 implementation [64] in paml [65]. We calculated pairwise SNPs in the intergenic alignment and calculated dI by dividing the number of SNPs by the length of the alignment. The dS values calculated from the core-gene alignment were used to calculate dN/dS and dI/dS.

## Results

### Pangenome and phylogenetic analyses

We *de novo* assembled and analysed core-genome alignments of 200 clinical *M. abscessus* (120 subsp. *abscessus* and 80 subsp. *massiliense*) isolates from seven geographical locations: Australia, Denmark, Dublin, Holland, Norway, the UK and the USA. Pangenome analysis of these 200 *M *. *abscessus* isolates defined a large accessory genome in both *M. abscessus* subsp. *abscessus* and subsp. *massiliense*, with many genes found at low frequencies. The core genome consisted of 3672 genes present in at least 99 % of isolates, whereas a total of 41 939 genes were found at lower frequencies (Table S2). The mean number of genes per isolate was comparable between *M. abscessus* subsp. *abscessus* and subsp. *massiliense*: 5182 and 5107, respectively.

**Fig. 1.**
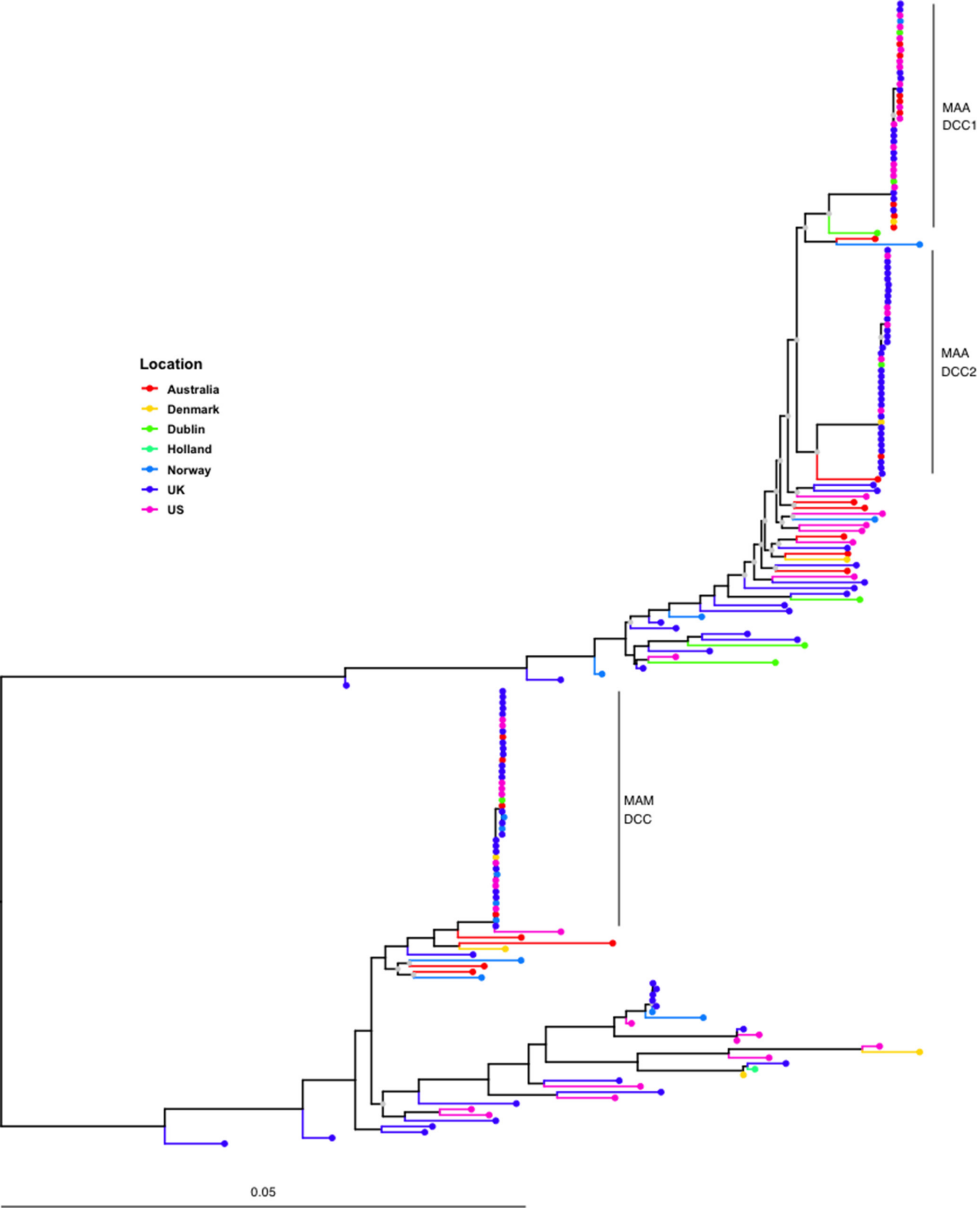
*
M. abscessus* subsp. *abscessus* (MAA) and subsp. *massiliense* (MAM) core-genome maximum-likelihood phylogeny shows distinct subspecies structure. We inferred a maximum-likelihood phylogeny from a core-genome alignment of 120 *M. abscessus* subsp. *abscessus* (top) and 80 *M. abscessus* subsp. *massiliense* (bottom) isolates. DCCs as defined by Bryant and colleagues [8] are labelled in both subspecies. Non-DCC isolates are considered EAIs. Isolates from different geographical regions are interspersed in the phylogeny, consistent with efficient global dispersal of bacteria. The phylogeny is midpoint rooted, and nodes with bootstrap values lower than 70 are labelled in grey. Branch lengths are scaled by the number of substitutions per site.

**Fig. 2.**
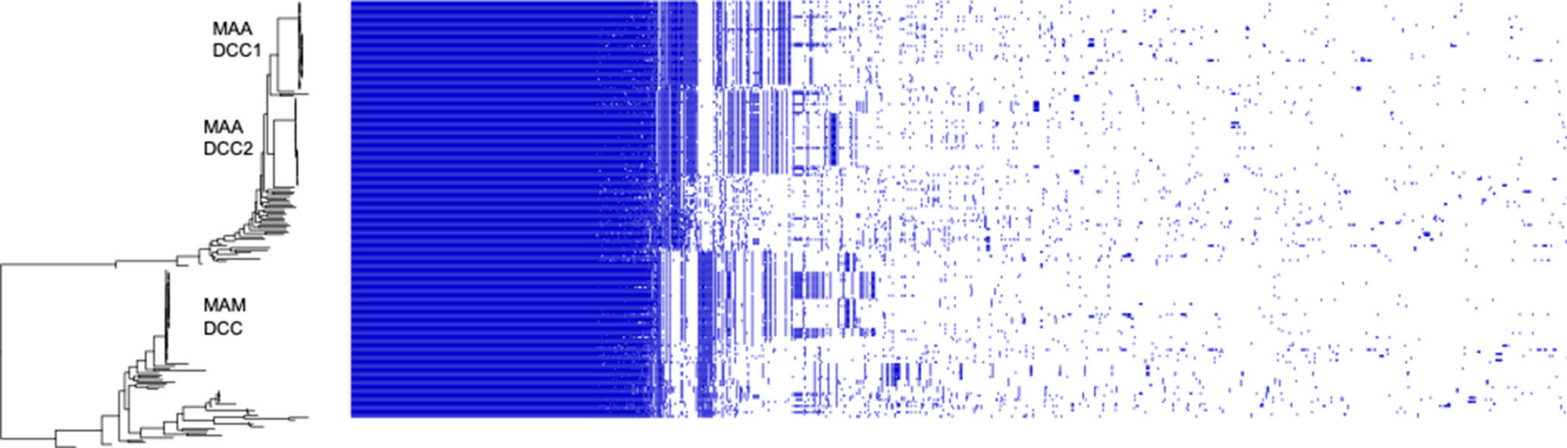
Accessory gene content of *M. abscessus* is structured by subspecies/clade. Gene homologues with 95 % amino acid similarity identified using Roary are plotted, excluding singleton genes. Order of species in the phylogenetic tree (left) corresponds to the species order in Fig. 1. Accessory content differs among *M. abscessus* subspecies and niche specialists (DCCs). Plot created using Phandango [44]. MAA, *M. abscessus* subsp. *abscessus *; MAM, *M. abscessus* subsp. *massiliense *.

We found the core genomes of *M. abscessus* subsp. *abscessus* and subsp. *massiliense* to be clearly differentiated, as shown by the long branch separating them in the maximum-likelihood phylogeny and network (Figs 1 and S1). This was also true of the accessory genome, with each subspecies characterized by distinct accessory gene content (Fig. 2). Both subspecies are globally distributed, with little evidence of geographical structure in either the host-associated DCCs or EAIs (Figs 1 and S2). The sample of *M. abscessus* subsp. *abscessus* includes two DCC clades, whereas subsp. *massiliense* contains one DCC clade (Figs 1 and S2). The DCCs of both species have larger genomes than their EAI counterparts: DCCs had a median gene count of 250, 200 and 150 genes higher than EAIs for the *M. abscessus* subsp. *abscessus* DCC1, DCC2 and *M. abscessus* subsp. *massiliense* DCC, respectively.

### LGT in the core genome

**Fig. 3.**
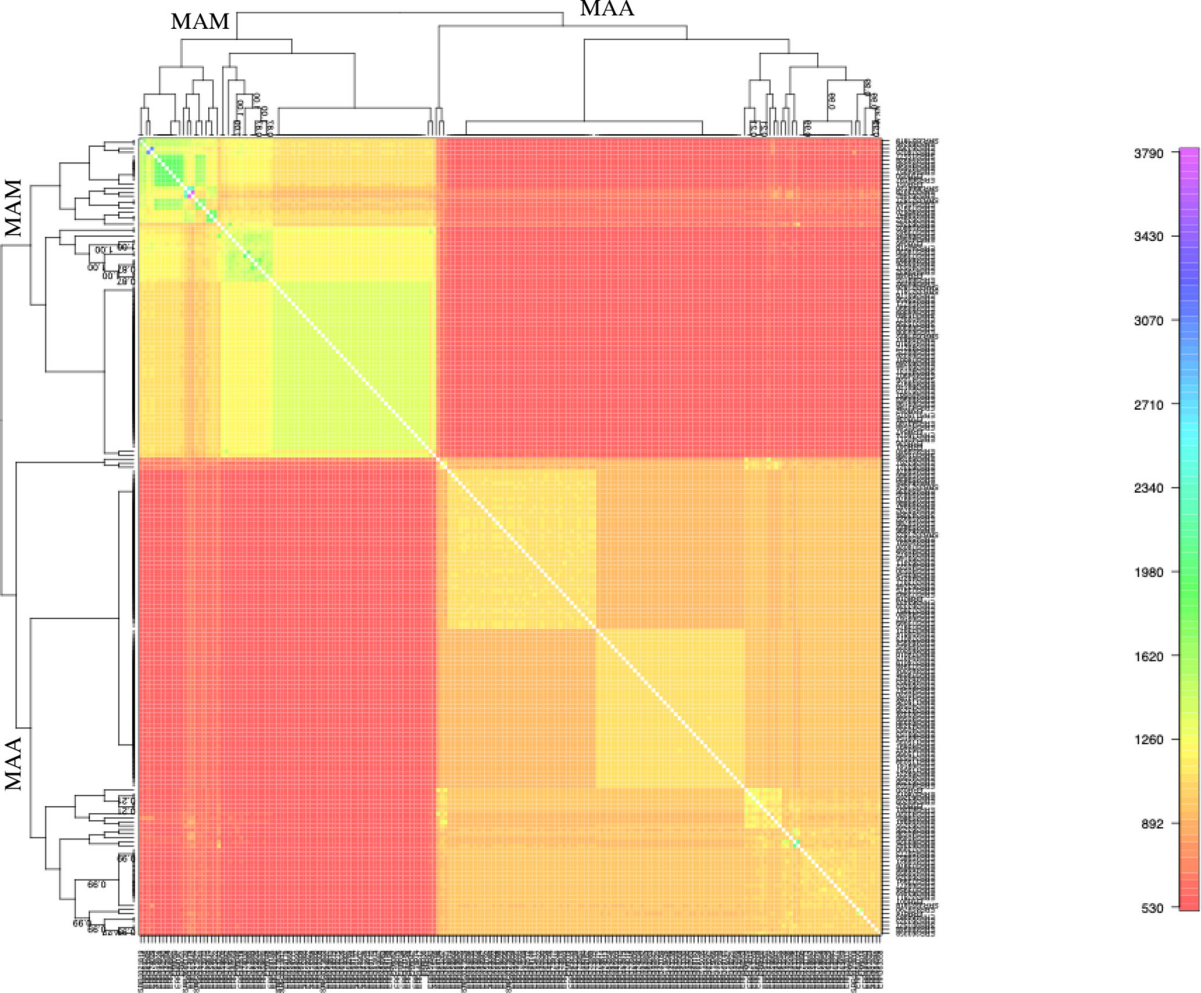
Co-ancestry matrix depicting population structure and gene flow in the core genome of 120 *M *. *abscessus* isolates. The co-ancestry matrix is coloured by the estimated fractions of genome fragments that are imported from a donor genome (column) to a recipient genome (row). For example, colours higher on the relative scale (right) indicate a higher proportion of transferred fragments. The trees on the top and left show clustering assignments as determined by FineSTRUCTURE. *M *. *abscessus* subsp. *massiliense* (MAM) and subsp. *abscessus* (MAA) appear genetically isolated as indicated by the large red rectangles in the matrix. Both *M *. *abscessus* subsp. *massiliense* (upper left) and subsp. *abscessus* (lower right) appear to recombine more frequently within subspecies than between. Within subspecies recombination appears more frequent in *M *. *abscessus* subsp. *massiliense* than in subsp. *abscessus*, indicated by the lighter yellow colour of the upper left versus lower right box.

In our analyses of LGT in the core genome of *M. abscessus *, we found little evidence of admixture between the two subspecies. A previous study using multilocus sequence typing data and structure [66] hypothesized that *M. abscessus* subspecies are admixed [67]; however, using FineSTRUCTURE [54], we found evidence for substantial genetic exchange within *M. abscessus* subsp. *abscessus* and subsp. *massiliense*, but limited admixture between them (Fig. 3).

**Fig. 4.**
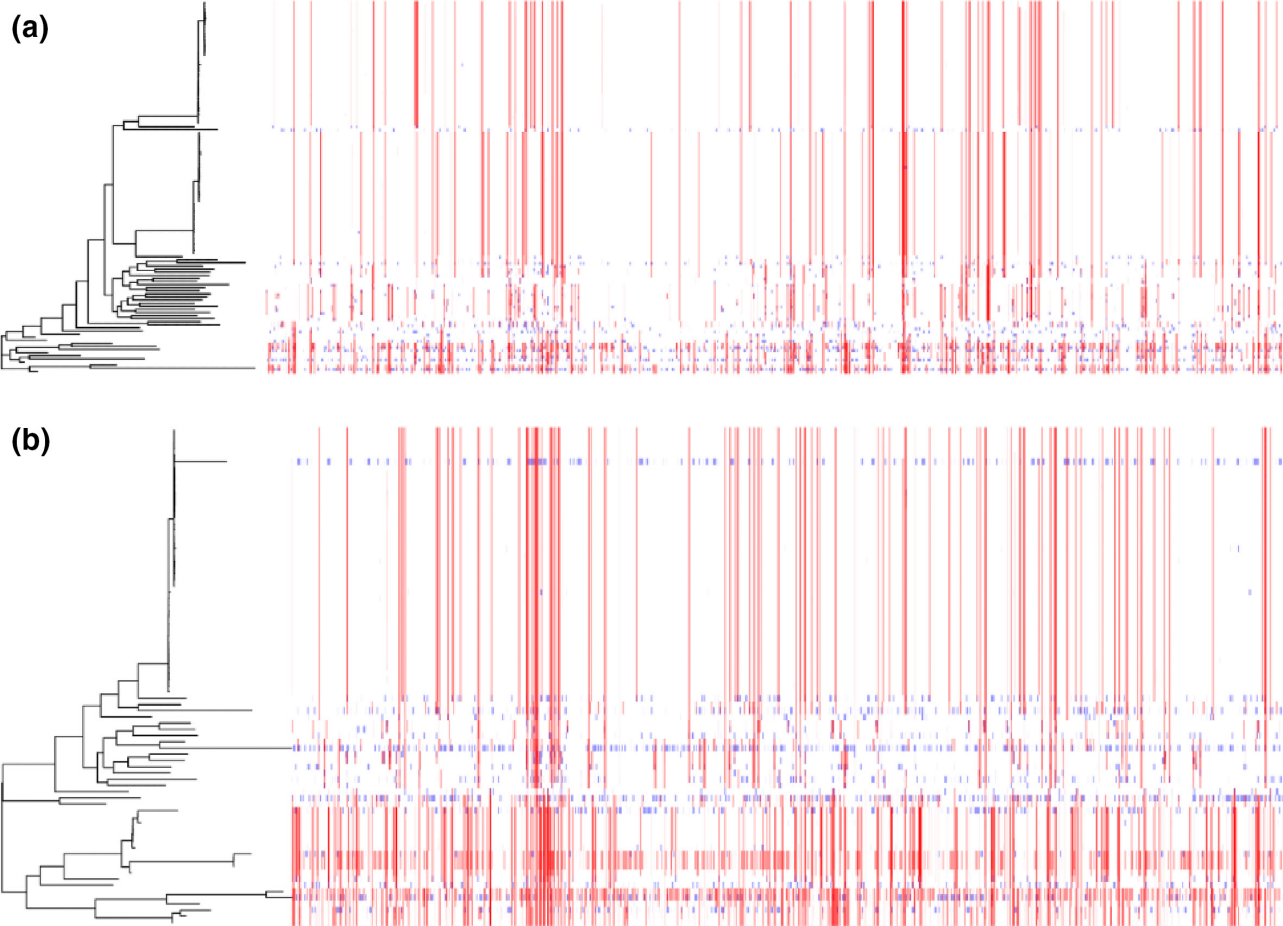
Within subspecies recombination is frequent in *M. abscessus* core genomes. Recombination in the core genomes of (**a**) *M. abscessus* subsp. *abscessus* and (**b**) subsp. *massiliense*. Each row corresponds to the core genome of an isolate in the phylogenetic tree to the left. Blue segments represent laterally transferred fragments unique to an individual isolate. Red segments indicate laterally transferred fragments that are shared across multiple isolates. The proportion of sites affected by recombination is 90.9 % for *M. abscessus* subsp. *abscessus* and 98.6 % for subsp. *massiliense*. The DCCs in both subspecies appear to have less recombination than EAIs.

**Fig. 5.**
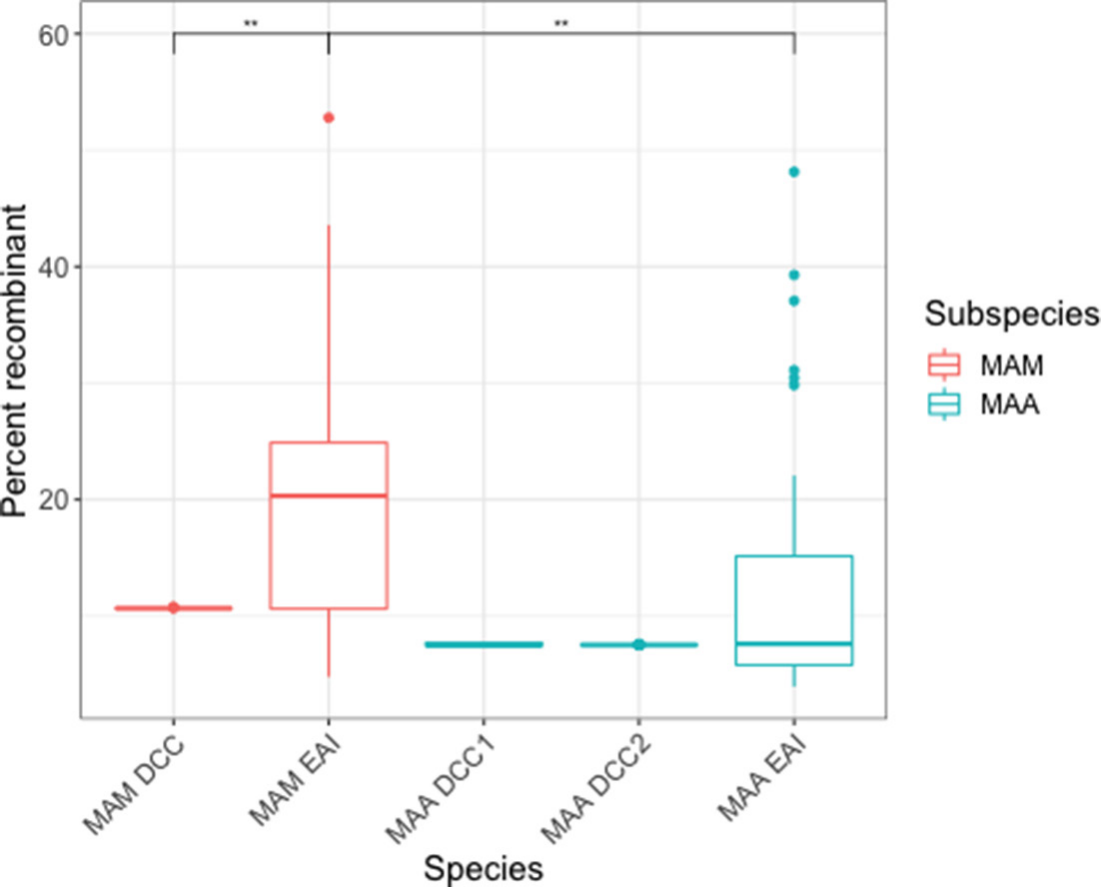
*
M. abscessus* subsp. *massiliense* (MAM) core genomes have more evidence of recombination than subsp. *abscessus* (MAA). Boxplots show the proportion of each isolate’s core genome affected by recombination, as estimated with Gubbins. The box spans the interquartile range, the median is represented by the middle line and the whiskers extend to ±1.5 times the interquartile range. Data beyond the end of the whiskers are outliers and plotted individually. The mean proportion of recombinant sites differs significantly by group (Kruskal–Wallis test, H=76, *P*<0.0001). The distribution of affected core genomes in *M. abscessus* subsp. *massiliense* EAIs is higher than that of *M. abscessus* subsp. *abscessus* EAIs (Mann–Whitney–Wilcoxon test, W=435, *P*=0.0045). Additionally, the *M. abscessus* subsp. *massiliense* DCCs have a significantly lower proportion of their genomes affected by recombination when compared to the *M. abscessus* subsp. *massiliense* EAI (Mann–Whitney–Wilcoxon test, W=435, *P*=0.0045). Finally, the DCCs from both subspecies have narrower distributions of proportion of the core genome affected by recombination than the EAIs. This is consistent with a reduction in recombination. *P* value: **, <0.01.

Based on these results, we characterized LGT in the core genomes of the two subspecies separately. We found more reticulations in the network of *M *. *abscessus* subsp. *massiliense *’s core genome than that of *M *. *abscessus* subsp. *abscessus* (Fig. S3), suggesting *M *. *abscessus* subsp. *massiliense* participates in LGT more than *M *. *abscessus* subsp. *abscessus *. Both subspecies did, however, show evidence of recombination (PHI (pairwise homoplasy index) test of recombination, *P*=0). Pangenomes of the two subspecies are of similar size (Fig. S4). Using Gubbins [43], we found that 99 % of sites in a core-genome alignment of *M *. *abscessus* subsp. *massiliense* has been affected by recombination, which compares to 91 % for *M *. *abscessus* subsp. *abscessus* (Fig. 4). In a pairwise comparison of EAIs, the mean proportion of recombinant sites was higher in *M *. *abscessus* subsp. *massiliense* than subsp. *abscessus* (Mann–Whitney–Wilcoxon test, W=435, *P*=0.0045) (Fig. 5). We found the mean size of recombinant tracts for *M. abscessus* subsp. *abscessus* and subsp. *massiliense* to be 3606 and 3252 bp, respectively. The distributions of recombinant fragment lengths for both *M. abscessus* subsp. *abscessus* and subsp. *massiliense* appear similar to that of *Mycobacterium canettii*, a species known to participate in DCT [48, 49] (Fig. S5). DCT is novel form of LGT that involves the transfer of multiple segments of chromosomal DNA from donor to recipient, and has thus far only been described in mycobacteria [68, 69].

In our analyses of LGT in the core genomes of *M. abscessus* subsp. *abscessus* and subsp. *massiliense*, we found differences in the patterns of LGT between the DCCs and EAIs. DCC isolates were more likely to have recombinant tracts shared across the entire clade (Figs. 4 and S6). Additionally, in the co-ancestry matrices of both subspecies, the DCCs are highly structured compared to EAIs, and appear less likely to recombine (Fig. S7). Across both subspecies, the proportion of recombinant sites is higher in EAIs than DCCs (Kruskal–Wallis test, H=76, *P*<0.01) (Fig. 5).

### Patterns of LGT in the accessory genome of *M. abscessus*

**Fig. 6.**
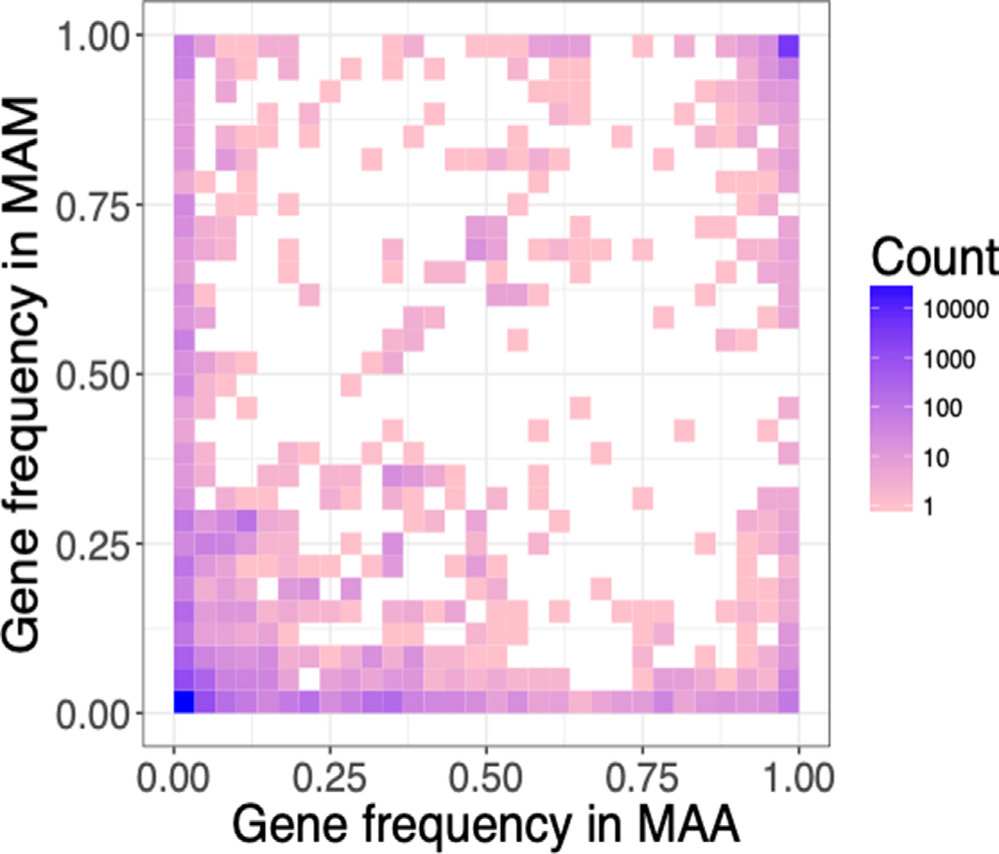
Shared accessory genes are found at different frequencies in *M. abscessus* subspecies. Heat map of gene frequencies in *M. abscessus* subsp. *abscessus* (MAA) and subsp. *massiliense* (MAM). Much of the accessory gene content is unique to each subspecies and is rare within subspecies. Shared accessory genes are maintained at distinct frequencies across the subspecies. This suggests there are barriers to gene flow as we would expect frequencies to equalize in the presence of free flow between subspecies.

When comparing the accessory genomes of *M. abscessus* subsp. *abscessus* and subsp. *massiliense*, we found several lines of evidence suggesting there are barriers to accessory gene flow between subspecies. First, shared accessory genes are maintained at different frequencies between the two subspecies (Fig. 6). In the setting of frequent exchange between subspecies, we would instead expect equalization of their accessory gene frequencies. If the two subspecies were readily exchanging their accessory genes, we would further expect intergroup measures of diversity (π) to be similar to measures within subspecies. Instead, we found genewise π values of shared accessory genes to be lower within subspecies than between subspecies (Kruskal–Wallace test, *P*<0.01; Mann–Whitney–Wilcoxon test, *P*<0.01) (Fig. S8). These results suggest accessory genes are transferred more frequently within than between subspecies.

**Fig. 7.**
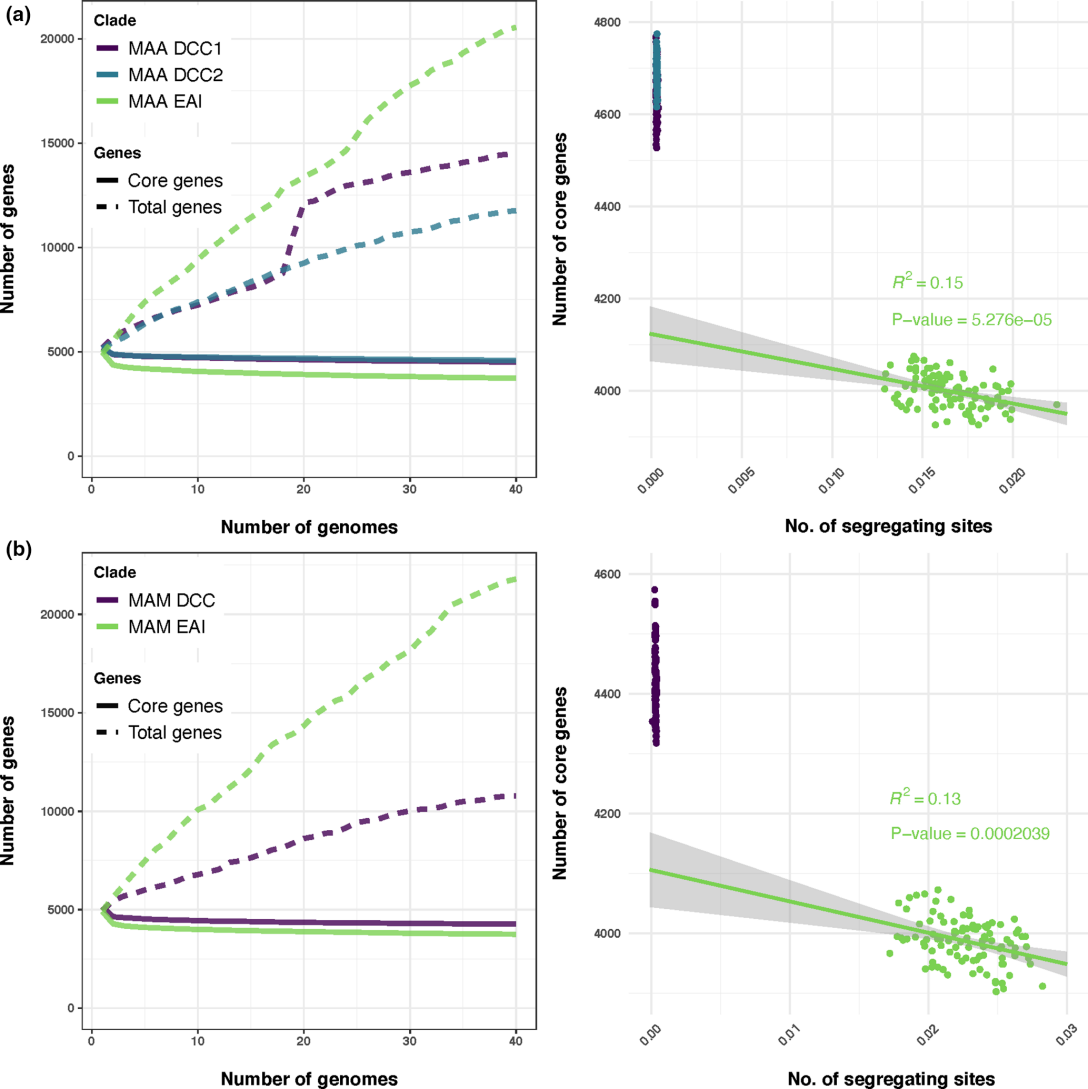
*
M. abscessus* EAIs have a larger pangenome than DCCs. In both *M. abscessus* subsp. *abscessus* (MAA) (**a**) and subsp. *massiliense* (MAM) (**b**), rarefaction plots show that EAIs have a larger pangenome and a smaller core genome than DCCs, consistent with lower rates of LGT among EAIs relative to DCCs. However, it is also possible that pangenome size reflects the timescales over which samples diverged, with the DCCs simply having had less time to accumulate novel genes. To test for this, we randomly sampled the EAIs, measured segregating sites as a proxy for divergence times and calculated the core-genome and pangenome sizes. Pangenome size was not significantly correlated with sample divergence (Fig. S9), whereas core-genome size was modestly correlated (right panels). The DCCs were outliers with respect to core-genome size, suggesting that observed trends are not fully accounted for by differences in sample divergence times.

DCCs and EAIs differed with respect to their pangenomes. In both subspecies, the pangenome is smaller and the core genome is larger for DCCs than for EAIs (Fig. 7). This is consistent with lower rates of LGT among DCCs relative to EAIs. However, given that DCCs are recently emerged, it is also possible that underlying rates of LGT are similar between groups but the DCCs have had less time to acquire novel gene content. In order to further investigate these alternatives, we assayed the relationships between proxy measures of divergence time, accessory-genome differentiation and pangenome expansion. We used segregating sites for the subspecies core-genome alignment as a proxy for divergence time of a sample. There was no significant correlation between divergence time and the overall size of the pangenome in either *M. abscessus* subsp. *massiliense* or subsp. *abscessus* (*R* ^2^ 0.03 and 0.04, respectively; *P*>0.05 for both) (Fig. S9), suggesting that the *M. abscessus* pangenome does not expand linearly as a sample diversifies. There was a significant correlation between core-genome size and sample diversification for both subspecies (*R* ^2^ 0.13 for *M. abscessus* subsp. *massiliense *, 0.15 for *M. abscessus* subsp. *abscessus *; *P*<0.001 for both) (Fig. 7). In the context of these trends, DCCs in both subspecies remained clear outliers with respect to the size of their core genomes (Fig. 7); that is, their core genomes appear larger than expected given the divergence time of the sample.

We also examined the relationship between core- and accessory-genome diversification at the level of individual isolates (Fig. S10). Here, we compared tree distances from the core-genome phylogeny with accessory-genome distances inferred from a gene presence/absence matrix for pairs of EAIs. Examining pairs of isolates in this way, we observed a linear relationship between core- and accessory-genome distances for EAIs from both subspecies (*R* ^2^ 0.24 for *M. abscessus s* ubsp. *massiliense *, 0.21 for subsp. *abscessus*; *P*<0.01 for both) suggesting that accessory-genome differentiation occurs in parallel with differentiation of the core genome. Synthesizing this observation with findings at the sample level, we infer that rates of acquisition of novel genes parallel rates of core-genome diversification, but that the pool of novel genes for EAIs is very large and unstructured such that the overall size of the pangenome does not expand linearly as a sample diversifies. We also analysed EAI–DCC pairs (Fig. S10) and found that for a given core-genome distance, accessory gene content of DCCs is more differentiated from EAIs than EAIs are from each other. This is consistent with genetic isolation of DCCs. Taken together, these results suggest that the pangenome dynamics of the DCCs are distinct from those of the EAIs, likely due to the DCCs losing access to the large pool of accessory genes shared among EAIs.

### Prophage analyses

**Fig. 8.**
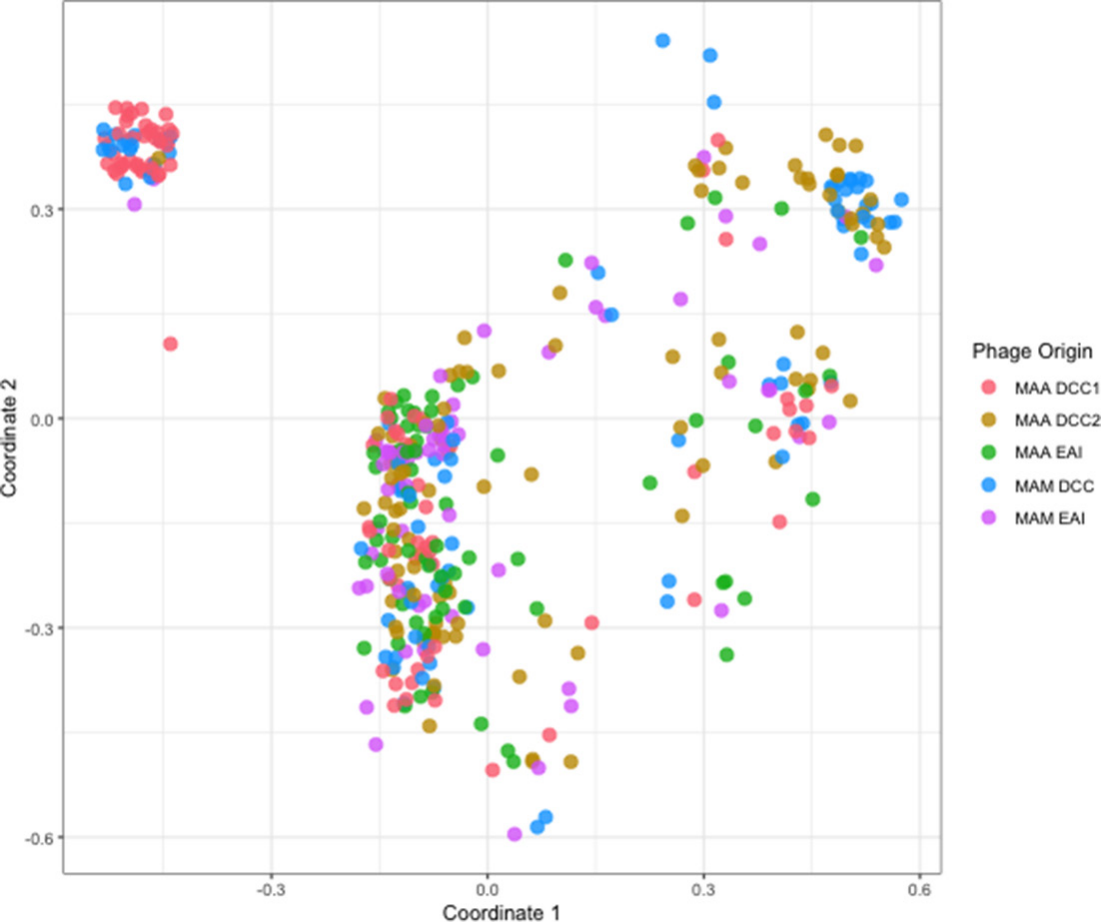
*
M. abscessus* prophage are readily exchanged between subspecies and strain types. We used multi-dimensional scaling of pairwise mash distances of prophage nucleotide sequences identified using ProphET to visualize prophage diversity and distribution across groups. Prophage do not cluster according to subspecies or strain type (DCC vs EAI), suggesting they are readily transferred between groups. Plot points jittered ±0.05 for greater visibility. MAA, *M. abscessus* subsp. *abscessus *; MAM, *M. abscessus* subsp. *massiliense *.

Despite barriers to LGT evident in the accessory genome, mobile genetic elements appear to be readily exchanged between subspecies. Using ProphET [58], we identified 407 prophage in our dataset of 200 *M *. *abscessus* genomes. We then used multi-dimensional scaling to visualize pairwise mash distances of these prophage and found that prophage from different bacterial groupings (DCC vs EAI, *M *. *abscessus* subsp. *abscessus* vs *s*ubsp. *massiliense*) were intermingled (Fig. 8). This suggests that prophage are readily transferred among otherwise genetically distinct isolates of *M. abscessus *.

### Genomic signatures of pathogen emergence

In addition to differences in recombination, we identified other genomic differences between DCCs and EAIs. *M *. *abscessus* subsp. *abscessus* DCC1 shows evidence of remodelling of its core genome relative to EAIs, with both loss of what was previously core gene content and gain of unique core gene content. *M *. *abscessus* subsp. *abscessus* DCC2 shows evidence of gain only, whereas *s*ubsp. *massiliense* DCC exhibits neither loss nor gain (Fig. S11). This suggests that, relative to the *M. abscessus s* ubsp. *massiliense* DCCs, the *M *. *abscessus* subsp. *abscessus* DCC may be further along an adaptive trajectory away from the EAIs and toward host association and specialization.

**Fig. 9.**
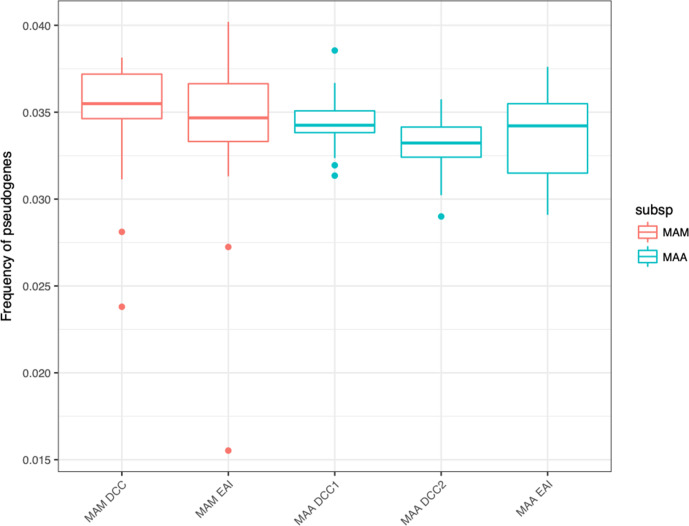
Levels of pseudogenization are low and similar between DCCs and EAIs. Counts of pseudogenes per isolate as a per cent of total intact genes per isolate are shown. We aligned all IGRs and blasted them against a custom *M. abscessus* proteome database. Pseudogenes were identified based on a published *E* value cut-off of 10e−5 [62]. *M *. *abscessus* subsp. *massiliense* (MAM) DCC and *M *. *abscessus* subsp. *abscessus* (MAA) DCC1 have slightly more pseudogenes than their respective EAIs, a difference that is not statistically significant.

The genomes of bacterial pathogens are often observed to be smaller relative to their free-living relatives and it has been posited that ‘genome downsizing’ is a feature of host adaptation [24, 25]. Pseudogenization may signal genome downsizing as newly unnecessary genes accumulate nonsense and other mutations that disrupt protein coding. We investigated the extent of pseudogenization among DCCs by identifying gene fragments using a blast method described previously [62]. The data did not show a consistent trend (Fig. 9) toward pseudogenization among the putatively host-adapted bacteria.

In order to investigate potential differences in selection pressures acting on DCCs and EAIs, we compared the distributions of pairwise πN/πS values across their core and accessory genomes. Gene-wise πN/πS values were higher in DCCs than in EAIs, for both subspecies (* M *. *abscessus* subsp. *abscessus* DCC1 vs EAI: Mann–Whitney–Wilcoxon test, W=2.9e5, *P*=0.0003) (Fig. S12). This suggests DCCs could be evolving under relaxed purifying selection relative to EAIs.

### Genetic diversity across IGRs

**Fig. 10.**
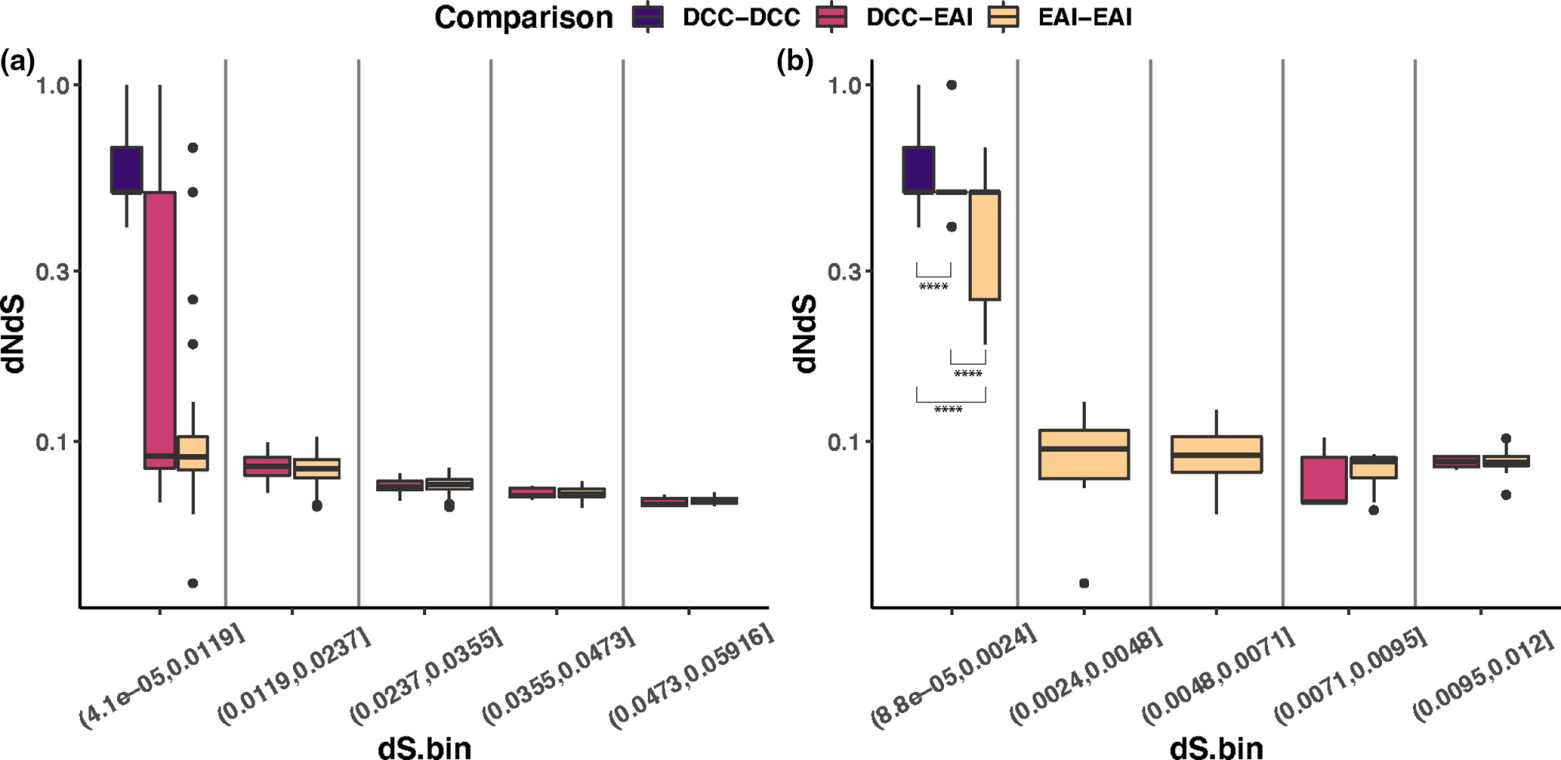
DCCs exhibit limited diversity with a skew to non-synonymous variation. (a) To investigate whether high values of dN/dS among DCCs could be explained by their relatively short divergence times, we plotted dN/dS for different values of dS. (b) The detail for the lowest values of dS. As expected, dN/dS values decrease as dS increases. However, DCCs exhibit higher than expected values of dN/dS relative to EAIs at the same depth of divergence (Mann–Whitney–Wilcoxon test, **** = *P* <0.001), consistent with relaxation of purifying selection.

**Fig. 11.**
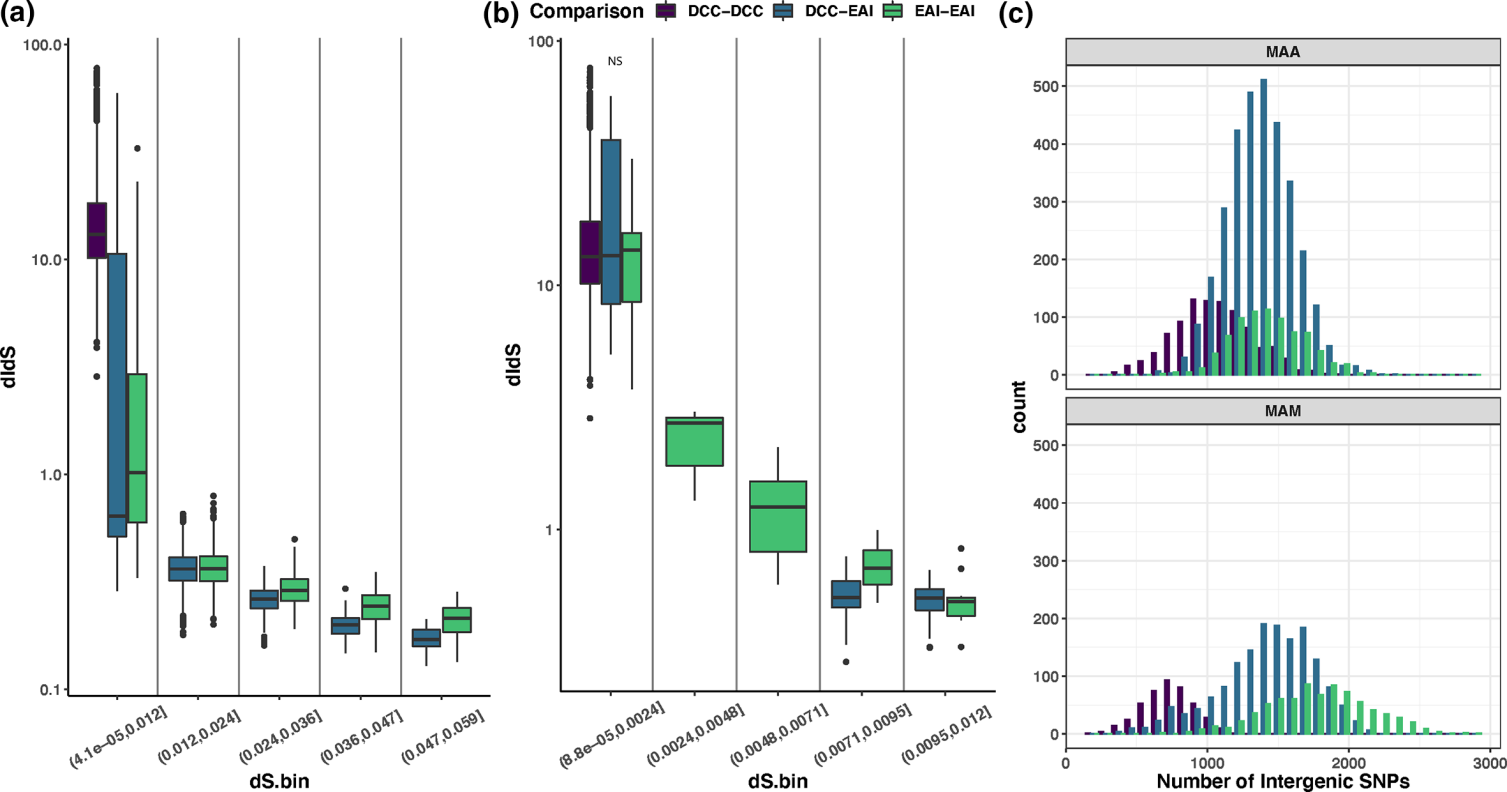
High rates of intergenic variation in DCCs due to recent emergence. (a) We calculated pairwise dI/dS values along intergenic alignments for each species and plotted dI/dS values across binned values of dS. (b) The detail for the lowest values of dS. NS = not significant. (c) Histograms of the number of intergenic SNPs in each pairwise comparison. For *M. abscessus* subsp. *massiliense* (MAM), the three distributions are distinct and ordered DCC–DCC, DCC–EAI and finally EAI–EAI; by contrast the three distributions are approximated in *M. abscessus* subsp. *abscessus* (MAA) indicating accumulation of intergenic SNPs in *M. abscessus* subsp. *abscessus* DCCs, consistent with maturation of the clone and remodelling of its core genome.

We hypothesized that the IGRs of the DCCs may be under distinct selection pressures relative to EAIs. To investigate this hypothesis, we calculated pairwise dI/dS and dN/dS values within and between groups for each subspecies. Distributions of dN/dS values were consistently lower than those of dI/dS, consistent with genic regions being under relatively strong selective constraint (Figs 10 and 11). Distributions of pairwise intergenic variants are closely approximated for DCC–DCC, DCC–EAI and EAI–EAI in *M *. *abscessus* subsp. *abscessus* and less so for *M *. *abscessus* subsp. *massiliense* (Fig. 11c). This is consistent with *M *. *abscessus* subsp. *abscessus* DCCs being further along in their emergence as reflected in the accumulation of intergenic variants, parallel to the observation of more extensive gene content remodelling in the core genome (Fig. S11).

### Impact of divergence time on patterns of variation

Divergence time has been shown to affect overall patterns of genetic diversity, with a greater proportion of variation at non-synonymous sites observed among recently diverged bacteria [70]. We hypothesized that this phenomenon could potentially explain differences observed between DCCs and EAIs. Measures of nearly neutral variation, i.e. at synonymous sites (dS), are lower among DCCs than EAIs, consistent with recent emergence and shorter divergence times (Figs 10 and 11). As expected, we observed non-synonymous variation to decrease as dS increased among EAIs (Fig. 10a). Interestingly, we observed an even stronger trend at intergenic sites (Fig. 11a), suggesting that many intergenic mutations are also deleterious and are removed over time. Relatively short divergence times appear to completely explain the high rates of intergenic variation observed among DCCs (Fig. 11b). However, this was not the case for genic variants, where there appears to be relaxation of purifying selection among DCCs relative to EAIs (Fig. 10b).

## Discussion

### *M. abscessus*: an emerging health threat

*M. abscessus* is a rapid growing, free-living mycobacterium that is capable of causing serious infections, particularly in people with CF and other lung diseases, as well as immunocompromised individuals [1, 3, 4]. The incidence of infection with non-tuberculous mycobacteria (NTMs) including *M. abscessus* has increased substantially over the past decade [71–73]. Treatment of *M. abscessus* infections is challenging due to both intrinsic and acquired resistance to antimicrobials [5–7]. The adaptability of this organism is further highlighted by the recent discovery of DCCs with evidence of person-to-person transmission [8–11].

### Mechanisms of LGT in *M. abscessus*

Our analyses point to what is likely to be an important source of this plasticity, namely the large pangenome and frequent LGT evident among *M. abscessus* (Figs 4, 5 and 7, Table S2). These findings are consistent with prior studies that predicted an open pangenome [74] and found evidence of LGT among *M. abscessus* [13, 67]. LGT in *M. abscessus* is likely to occur via multiple mechanisms. We identified abundant prophage in our sample, consistent with previous findings [16]. The distribution and relatedness of these prophage suggest that active transduction is occurring across subspecies (Fig. 8). We did not attempt to identify plasmids, as short-read sequencing data are unreliable for this purpose [75]. Plasmids are likely to be another mechanism of LGT, as previous studies have identified conjugative plasmids in *M. abscessus* [8, 14–16].

DCT is another mechanism of LGT that has so far been uniquely identified in mycobacteria [50, 51]. Genetic requirements for DCT in *M. smegmatis* include the type VII secretion system (T7SS) loci ESX-1 and ESX-4 [76–78]. *M. abscessus* encodes T7SS loci ESX-4 or ESX-4-bis/_EVOL_ and ESX-3, as well as plasmids containing T7SS loci [14, 16, 79]. Our analyses suggest that *M. abscessus* engages in DCT, as we found recombinant fragments in the core genome to be similar in length to those of *M. canettii*, which is known to engage in DCT (Fig. S6) [48, 49]. Long recombinant fragment lengths are a characteristic feature of DCT [48, 68]. Taken together, our results suggest that LGT occurs via multiple mechanisms in *M. abscessus *.

### Barriers to LGT between *M. abscessus* subspecies

A previous study found evidence suggesting that *M. abscessus s* ubsp. *massiliense* engages in LGT more frequently than does subsp. *abscessus* [13]. Our results support and extend these observations: we found the proportion of sites affected by recombination in the EAIs to be higher in *M. abscessus s* ubsp. *massiliense* than subsp. *abscessus* (Figs. 4 and 5), and the pace of accessory-genome diversification to be higher in *M. abscessus s* ubsp. *massiliense* relative to subsp. *abscessus* (Fig. S9). A previous report of whole-genome sequence data from three isolates mapped to different references interpreted mapping patterns as indicative of admixture between subspecies of *M. abscessus* and hypothesized that this was the result of DCT between subspecies [67]. DCT is likely to require substantial sequence similarity between donor and recipient genomes [68]. We found the core genomes of *M. abscessus s* ubsp. *massiliense* and subsp. *abscessus* to be genetically distinct, without evidence of substantial admixture (Fig. 3); we further found several lines of evidence indicating there are barriers to LGT between subspecies’ accessory genomes (Figs. 6 and S8). Prophage are an exception, as they appear to be readily shared across subspecies (Fig. 8). These findings indicate that while LGT is frequent among *M. abscessus *, this exchange is organized such that bacterial population structure is maintained. We recently identified similar phenomena among the highly recombinogenic *Gardnerella* species [80]. This bacterial population structure could reflect niche partitioning that maintains functional differences between sub-populations, and/or mechanical barriers to LGT, e.g. in transfer via DCT or plasmid conjugation.

### Genomic signatures of pathogen emergence in *M. abscessus*

*M. abscessus* infection was previously thought to be exclusively acquired from the environment; however, multiple globally distributed DCCs were recently identified that appear to be transmitted person to person among people with CF [8] (Fig. 1). The DCCs are illustrative of the genomic changes that accompany host adaptation and bacterial pathogen emergence. Prior research has identified pseudogenization, genome downsizing and a loss of metabolic capacity in comparisons of host-adapted with free-living bacteria [24–27, 81]. A recently published study of *M. abscessus* [82] found that some functional categories were enriched among accessory gene content acquired by DCCs. We found putatively host-adapted DCCs to be characterized by lower levels of LGT relative to environmentally acquired strains of *M. abscessus* (Figs. 4, 5 and 7). This echoes observations of the highly pathogenic species *M. tuberculosis *: while there is evidence of frequent LGT in its closest relative, *M. canettii*, *M. tuberculosis* appears to evolve strictly clonally [48, 49]. *M. tuberculosis* is transmitted person to person, whereas *M. canettii* is thought to be acquired from the environment or another reservoir [83]. Similar patterns have been observed outside the mycobacteria, e.g. in comparisons of pathogenic and non-pathogenic strains of *Streptococcus sui* s [84]. Free-living *M. abscessus* encode a massive pangenome (Fig. 7), which we hypothesize is fuelled by LGT with diverse partners found in soil and similarly complex niches. We further hypothesize that the CF lung offers fewer opportunities to partner with diverse bacteria, such that the DCCs’ occupation of this niche is reflected in contraction of their pangenomes relative to the EAIs.

### IGR remodelling during pathogen emergence

Although IGRs have traditionally been neglected as targets of selection, more recent research has uncovered a rich array of functions in association with these loci [85–87] and methods have been developed to characterize selection pressures at IGRs [60]. IGRs have been identified as a target of positive selection in *M. tuberculosis* [63]. We hypothesized that regulatory elements could be involved in host adaptation of *M. abscessus* and that this would be reflected in elevated dI/dS. DCCs were indeed characterized by relatively high rates of variation at intergenic sites (Fig. 11a). Extrapolating from the literature on adaptation at genic sites [70], we investigated whether intergenic variants appear to be pruned as bacteria diverge from each other. Our data are consistent with this phenomenon, as dI/dS decreased dramatically among the EAIs as dS increased (Fig. 11). This phenomenon appears to explain the high levels of intergenic variation observed among DCCs (Fig. 11b). Together, these data suggest that purifying selection is an important force shaping adaptation at intergenic sites in *M. abscessus *.

We found evidence of relaxation of purifying selection in the core and accessory genomes, reflected in elevated values of πN/πS relative to EAIs (Fig. S12). A recently published study [82] reported that the DCCs were under purifying selection based on dN/dS estimates <1 on the phylogenetic branch leading to the DCCs. It is difficult to interpret and contextualize their result as they used an unpublished method and did not compare DCCs with EAIs. Our findings are broadly consistent (πN/πS <1), but comparison with EAIs reveals a relative increase in non-synonymous variants among the DCCs. This likely reflects their recent emergence and divergence, and possibly linkage of mildly deleterious mutations to those positively selected during invasion of a new niche [70]. We did find that the increase in non-synonymous variation was out of keeping with divergence time, as measured by dS (Fig. 10b), suggesting that some protein-altering mutations have become newly tolerable during the shift to host adaptation. Interestingly, this pattern differs from observations at intergenic sites (Fig. 11b): we hypothesize that this could be due to greater pleiotropy of regulatory versus genic mutations. The distribution of dI/dS values among DCCs has a long tail (Fig. 11). High levels of intergenic mutations in some isolates could reflect cascades of mutations as regulatory mutations engender bursts of subsequent compensatory mutations that offset negative pleiotropy.

As noted above, pseudogenization has previously been identified in association with the shift to a host-associated lifestyle. We did not observe large-scale pseudogenization across the DCCs (Fig. 9). This is consistent with recently published research that found patterns of diversity among pathogens to be distinct from what had previously been described for other host-associated bacteria [84]. Murray *et al*. found that unlike symbionts, bacterial pathogen genomes were not characterized by excess pseudogenes or a loss of metabolic coding capacity.

### Conclusion

In conclusion, *M. abscessus* populations are characterized by a complex structure, within which subspecies maintain genetic differentiation in their core and accessory genomes despite overall high levels of LGT mediated by multiple mechanisms. Phage are an exception in that they appear to be readily exchanged among subspecies, suggesting that barriers between subspecies are not maintained by specific pairings of host and phage. Evolving niche specialists, the DCCs, show evidence of genome remodelling and a dramatic shift in their mode of evolution with less evidence of LGT. This could reflect a loss of opportunity to partner with diverse bacteria and/or loss of the capacity to perform this function. The core genomes of DCCs exhibit varying levels of core-genome remodelling, consistent with successive emergence and slightly different placement along an evolutionary trajectory toward host adaptation. The DCCs appear to be under relaxed purifying selection relative to their free-living relatives, as has been observed in other pathogenic bacterial species. Consistent with recent research contrasting pathogens with other host-associated bacteria, we did not identify large-scale pseudogenization among the DCCs. Taken together, these results provide insight into adaptation of this important pathogen and the genomic remodelling events that occur during emergence into a new niche.

## Supplementary Data

Supplementary material 1

## References

[1] Lopeman RC, Harrison J, Desai M, Cox JAG (2019). *Mycobacterium abscessus*: environmental bacterium turned clinical nightmare. Microorganisms.

[2] Johansen MD, Herrmann J-L, Kremer L (2020). Non-tuberculous mycobacteria and the rise of *Mycobacterium abscessus*. Nat Rev Microbiol.

[3] Moore M, Frerichs JB (1953). An unusual acid-fast infection of the knee with subcutaneous, abscess-like lesions of the gluteal region; report of a case with a study of the organism, *Mycobacterium abscessus*, n. sp. J Invest Dermatol.

[4] Floto RA, Olivier KN, Saiman L, Daley CL, Herrmann J-L (2016). US cystic fibrosis foundation and European cystic fibrosis society consensus recommendations for the management of non-tuberculous mycobacteria in individuals with cystic fibrosis: executive summary. Thorax.

[5] Griffith DE, Brown-Elliott BA, Benwill JL, Wallace RJ (2015). *Mycobacterium abscessus*. “Pleased to meet you, hope you guess my name…”. Ann Am Thorac Soc.

[6] Jarand J, Levin A, Zhang L, Huitt G, Mitchell JD (2011). Clinical and microbiologic outcomes in patients receiving treatment for *Mycobacterium abscessus* pulmonary disease. Clin Infect Dis.

[7] Choi H, Jhun BW, Kim S-Y, Kim DH, Lee H (2018). Treatment outcomes of macrolide-susceptible *Mycobacterium abscessus* lung disease. Diagn Microbiol Infect Dis.

[8] Bryant JM, Grogono DM, Rodriguez-Rincon D, Everall I, Brown KP (2016). Emergence and spread of a human-transmissible multidrug-resistant nontuberculous mycobacterium. Science.

[9] Bryant JM, Grogono DM, Greaves D, Foweraker J, Roddick I (2013). Whole-genome sequencing to identify transmission of *Mycobacterium abscessus* between patients with cystic fibrosis: a retrospective cohort study. Lancet.

[10] Davidson RM, Hasan NA, Epperson LE, Benoit JB, Kammlade SM (2021). Population genomics of *Mycobacterium abscessus* from United States cystic fibrosis care centers. Ann Am Thorac Soc.

[11] Doyle RM, Rubio M, Dixon G, Hartley J, Klein N (2020). Cross-transmission is not the source of new *Mycobacterium abscessus* infections in a multicenter cohort of cystic fibrosis patients. Clin Infect Dis.

[12] Tortoli E, Kohl TA, Brown-Elliott BA, Trovato A, Leão SC (2016). Emended description of *Mycobacterium abscessus*, *Mycobacterium abscessus* subsp*. abscessus* and *Mycobacterium abscessus* subsp. *bolletii* and designation of *Mycobacterium abscessus* subsp. *massiliense* comb. nov. Int J Syst Evol Microbiol.

[13] Tan JL, Ng KP, Ong CS, Ngeow YF (2017). Genomic comparisons reveal microevolutionary differences in *Mycobacterium abscessus* subspecies. Front Microbiol.

[14] Ripoll F, Pasek S, Schenowitz C, Dossat C, Barbe V (2009). Non mycobacterial virulence genes in the genome of the emerging pathogen *Mycobacterium abscessus*. PLoS One.

[15] Leão SC, Matsumoto CK, Carneiro A, Ramos RT, Nogueira CL (2013). The detection and sequencing of a broad-host-range conjugative IncP-1β plasmid in an epidemic strain of *Mycobacterium abscessus* subsp. *bolletii*. PLoS One.

[16] Dedrick RM, Aull HG, Jacobs-Sera D, Garlena RA, Russell DA (2021). The prophage and plasmid mobilome as a likely driver of *Mycobacterium abscessus* diversity. mBio.

[17] Gutierrez MC, Brisse S, Brosch R, Fabre M, Omaïs B (2005). Ancient origin and gene mosaicism of the progenitor of *Mycobacterium tuberculosis*. PLoS Pathog.

[18] Becq J, Gutierrez MC, Rosas-Magallanes V, Rauzier J, Gicquel B (2007). Contribution of horizontally acquired genomic islands to the evolution of the tubercle bacilli. Mol Biol Evol.

[19] Supply P, Marceau M, Mangenot S, Roche D, Rouanet C (2013). Genomic analysis of smooth tubercle bacilli provides insights into ancestry and pathoadaptation of *Mycobacterium tuberculosis*. Nat Genet.

[20] Sreevatsan S, Pan X, Stockbauer KE, Connell ND, Kreiswirth BN (1997). Restricted structural gene polymorphism in the *Mycobacterium tuberculosis* complex indicates evolutionarily recent global dissemination. Proc Natl Acad Sci USA.

[21] Hirsh AE, Tsolaki AG, DeRiemer K, Feldman MW, Small PM (2004). Stable association between strains of *Mycobacterium tuberculosis* and their human host populations. Proc Natl Acad Sci USA.

[22] Pepperell C, Hoeppner VH, Lipatov M, Wobeser W, Schoolnik GK (2010). Bacterial genetic signatures of human social phenomena among *M. tuberculosis* from an aboriginal Canadian population. Mol Biol Evol.

[23] Pepperell CS, Casto AM, Kitchen A, Granka JM, Cornejo OE (2013). The role of selection in shaping diversity of natural *M. tuberculosis* populations. PLoS Pathog.

[24] Murray GGR, Charlesworth J, Miller EL, Casey MJ, Lloyd CT (2021). Genome reduction is associated with bacterial pathogenicity across different scales of temporal and ecological divergence. Mol Biol Evol.

[25] Weinert LA, Welch JJ (2017). Why might bacterial pathogens have small genomes?. Trends Ecol Evol.

[26] Stinear TP (2008). Inights from the complete genome sequence of *Mycobacterium marinum* on the evolution of *Mycobacterium tuberculosis*. Genome Res.

[27] Ochman H (2001). Genes lost and genes found: evolution of bacterial pathogenesis and symbiosis. Science.

[28] Andrews S (2010). http://www.bioinformatics.babraham.ac.uk/projects/fastqc/.

[29] Krueger F (2015). https://www.bioinformatics.babraham.ac.uk/projects/trim_galore/.

[30] Li H, Handsaker B, Wysoker A, Fennell T, Ruan J (2009). The sequence alignment/map format and SAMtools. Bioinformatics.

[31] Walker BJ, Abeel T, Shea T, Priest M, Abouelliel A (2014). Pilon: an integrated tool for comprehensive microbial variant detection and genome assembly improvement. PLoS One.

[32] Price MN (2010). FastTree 2 – approximately maximum-likelihood trees for large alignments. PLoS One.

[33] Harris SR (2017). https://github.com/simonrharris/tree_gubbins.

[34] Menardo F, Loiseau C, Brites D, Coscolla M, Gygli SM (2018). Treemmer: a tool to reduce large phylogenetic datasets with minimal loss of diversity. BMC Bioinformatics.

[35] Eldholm V, Norheim G, von der Lippe B, Kinander W, Dahle UR (2014). Evolution of extensively drug-resistant *Mycobacterium tuberculosis* from a susceptible ancestor in a single patient. Genome Biol.

[36] Bankevich A, Nurk S, Antipov D, Gurevich AA, Dvorkin M (2012). SPAdes: a new genome assembly algorithm and its applications to single-cell sequencing. J Comput Biol.

[37] Seemann T (2014). Prokka: rapid prokaryotic genome annotation. Bioinformatics.

[38] Gurevich A, Saveliev V, Vyahhi N, Tesler G (2013). QUAST: quality assessment tool for genome assemblies. Bioinformatics.

[39] Page AJ, Cummins CA, Hunt M, Wong VK, Reuter S (2015). Roary: rapid large-scale prokaryote pan genome analysis. Bioinformatics.

[40] Löytynoja A (2014). Phylogeny-aware alignment with PRANK. Methods Mol Biol.

[41] Stamatakis A (2014). RAxML version 8: a tool for phylogenetic analysis and post-analysis of large phylogenies. Bioinformatics.

[42] Yu G, Smith DK, Zhu H, Guan Y, Lam TT (2016). ggtree: an R package for visualization and annotation of phylogenetic trees with their covariates and other associated data. Methods Ecol Evol.

[43] Croucher NJ, Page AJ, Connor TR, Delaney AJ, Keane JA (2015). Rapid phylogenetic analysis of large samples of recombinant bacterial whole genome sequences using Gubbins. Nucleic Acids Res.

[44] Hadfield J, Croucher NJ, Goater RJ, Abudahab K, Aanensen DM (2018). Phandango: an interactive viewer for bacterial population genomics. Bioinformatics.

[45] Kruskal WH, Wallis WA (1952). Use of ranks in one-criterion variance analysis. J Am Stat Assoc.

[46] Mann HB, Whitney DR (1947). On a test of whether one of two random variables is stochastically larger than the other. Ann Math Statist.

[47] Bonferroni CE (1935). Il calcolo delle assicurazioni su gruppi di teste. Studi Onore Profr Salvatore Ortu Carboni.

[48] Mortimer TD, Pepperell CS (2014). Genomic signatures of distributive conjugal transfer among mycobacteria. Genome Biol Evol.

[49] Boritsch EC, Khanna V, Pawlik A, Honoré N, Navas VH (2016). Key experimental evidence of chromosomal DNA transfer among selected tuberculosis-causing mycobacteria. Proc Natl Acad Sci USA.

[50] Parsons LM, Jankowski CS, Derbyshire KM (1998). Conjugal transfer of chromosomal DNA in *Mycobacterium smegmatis*. Mol Microbiol.

[51] Wang J, Derbyshire KM (2004). Plasmid DNA transfer in *Mycobacterium smegmatis* involves novel DNA rearrangements in the recipient, which can be exploited for molecular genetic studies. Mol Microbiol.

[52] Huson DH, Bryant D (2006). Application of phylogenetic networks in evolutionary studies. Mol Biol Evol.

[53] Bruen TC, Philippe H, Bryant D (2006). A simple and robust statistical test for detecting the presence of recombination. Genetics.

[54] Lawson DJ, Hellenthal G, Myers S, Falush D (2012). Inference of population structure using dense haplotype data. PLoS Genet.

[55] Page AJ, Taylor B, Delaney AJ, Soares J, Seemann T (2016). SNP-sites: rapid efficient extraction of SNPs from multi-FASTA alignments. Microb Genom.

[56] Danecek P, Auton A, Abecasis G, Albers CA, Banks E (2011). The variant call format and VCFtools. Bioinformatics.

[57] De Mita S, Siol M (2012). EggLib: processing, analysis and simulation tools for population genetics and genomics. BMC Genet.

[58] Reis-Cunha JL, Bartholomeu DC, Manson AL, Earl AM, Cerqueira GC (2019). ProphET, prophage estimation tool: a stand-alone prophage sequence prediction tool with self-updating reference database. PLoS One.

[59] Ondov BD, Treangen TJ, Melsted P, Mallonee AB, Bergman NH (2016). Mash: fast genome and metagenome distance estimation using MinHash. Genome Biol.

[60] Thorpe HA, Bayliss SC, Sheppard SK, Feil EJ (2018). Piggy: a rapid, large-scale pan-genome analysis tool for intergenic regions in bacteria. Gigascience.

[61] Altschul SF, Gish W, Miller W, Myers EW, Lipman DJ (1990). Basic local alignment search tool. J Mol Biol.

[62] Belda E, Moya A, Bentley S, Silva FJ (2010). Mobile genetic element proliferation and gene inactivation impact over the genome structure and metabolic capabilities of *Sodalis glossinidius*, the secondary endosymbiont of tsetse flies. BMC Genomics.

[63] Thorpe HA, Bayliss SC, Hurst LD, Feil EJ (2017). Comparative analyses of selection operating on nontranslated intergenic regions of diverse bacterial species. Genetics.

[64] Yang Z, Nielsen R (2000). Estimating synonymous and nonsynonymous substitution rates under realistic evolutionary models. Mol Biol Evol.

[65] Yang Z (1997). PAML: a program package for phylogenetic analysis by maximum likelihood. Comput Appl Biosci.

[66] Falush D, Stephens M, Pritchard JK (2007). Inference of population structure using multilocus genotype data: dominant markers and null alleles. Mol Ecol Notes.

[67] Sapriel G, Konjek J, Orgeur M, Bouri L, Frézal L (2016). Genome-wide mosaicism within *Mycobacterium abscessus*: evolutionary and epidemiological implications. BMC Genomics.

[68] Gray TA, Krywy JA, Harold J, Palumbo MJ, Derbyshire KM (2013). Distributive conjugal transfer in mycobacteria generates progeny with meiotic-like genome-wide mosaicism, allowing mapping of a mating identity locus. PLoS Biol.

[69] Gray TA, Derbyshire KM (2018). Blending genomes: distributive conjugal transfer in mycobacteria, a sexier form of HGT. Mol Microbiol.

[70] Rocha EPC, Smith JM, Hurst LD, Holden MTG, Cooper JE (2006). Comparisons of dN/dS are time dependent for closely related bacterial genomes. J Theor Biol.

[71] Bar-On O, Mussaffi H, Mei-Zahav M, Prais D, Steuer G (2015). Increasing nontuberculous mycobacteria infection in cystic fibrosis. J Cyst Fibros.

[72] Roux A-L, Catherinot E, Ripoll F, Soismier N, Macheras E (2009). Multicenter study of prevalence of nontuberculous mycobacteria in patients with cystic fibrosis in France. J Clin Microbiol.

[73] Olivier KN, Weber DJ, Wallace RJ, Faiz AR, Lee J-H (2003). Nontuberculous mycobacteria. Am J Respir Crit Care Med.

[74] Choo SW, Wee WY, Ngeow YF, Mitchell W, Tan JL (2014). Genomic reconnaissance of clinical isolates of emerging human pathogen *Mycobacterium abscessus* reveals high evolutionary potential. Sci Rep.

[75] Arredondo-Alonso S, Willems RJ, van Schaik W, Schürch AC (2017). On the (im)possibility of reconstructing plasmids from whole-genome short-read sequencing data. Microb Genom.

[76] Coros A, Callahan B, Battaglioli E, Derbyshire KM (2008). The specialized secretory apparatus ESX-1 is essential for DNA transfer in *Mycobacterium smegmatis*. Mol Microbiol.

[77] Flint JL, Kowalski JC, Karnati PK, Derbyshire KM (2004). The RD1 virulence locus of *Mycobacterium tuberculosis* regulates DNA transfer in *Mycobacterium smegmatis*. Proc Natl Acad Sci USA.

[78] Gray TA, Clark RR, Boucher N, Lapierre P, Smith C (2016). Intercellular communication and conjugation are mediated by ESX secretion systems in mycobacteria. Science.

[79] Mortimer TD, Weber AM, Pepperell CS (2017). Evolutionary thrift: mycobacteria repurpose plasmid diversity during adaptation of type VII secretion systems. Genome Biol Evol.

[80] Bohr LL, Mortimer TD, Pepperell CS (2020). Lateral gene transfer shapes diversity of *Gardnerella* spp. Front Cell Infect Microbiol.

[81] Georgiades K, Raoult D, El-Sayed N (2011). Genomes of the most dangerous epidemic bacteria have a virulence repertoire characterized by fewer genes but more toxin-antitoxin modules. PLoS One.

[82] Bryant JM, Brown KP, Burbaud S, Everall I, Belardinelli JM (2021). Stepwise pathogenic evolution of *Mycobacterium abscessus*. Science.

[83] Koeck J-L, Fabre M, Simon F, Daffé M, Garnotel E (2011). Clinical characteristics of the smooth tubercle bacilli ‘*Mycobacterium canettii*’ infection suggest the existence of an environmental reservoir. Clin Microbiol Infect.

[84] Murray GGR, Charlesworth J, Miller EL, Casey MJ, Lloyd CT (2021). Genome reduction is associated with bacterial pathogenicity across different scales of temporal and ecological divergence. Mol Biol Evol.

[85] Molina N, van Nimwegen E (2008). Universal patterns of purifying selection at noncoding positions in bacteria. Genome Res.

[86] Luo H, Tang J, Friedman R, Hughes AL (2011). Ongoing purifying selection on intergenic spacers in group A streptococcus. Infect Genet Evol.

[87] Degnan PH, Ochman H, Moran NA, Casadesús J (2011). Sequence conservation and functional constraint on intergenic spacers in reduced genomes of the obligate symbiont *Buchnera*. PLoS Genet.

