# Supplementary material for: Genome reorganization during emergence of host-associated Mycobacterium abscessus
Source: Microb Genom. 2021 Dec 7;7(12):000706. doi: 10.1099/mgen.0.000706 (PMC8767326; doi:10.1099/mgen.0.000706)
Supplement: Supplementary material 1 [file mgen-7-0706-s001.pdf]

Supplemental Materials

Table S1. Strain accession numbers and pertinent metadata for all 120 *M. abscessus* isolates in this study. *indicates newly deposited isolates.

StrainName	SRA	StrainType	Country	Subspecies
ERR363358	ERR363358	EAI	UK	MAA
ERR363325	ERR363325	EAI	Dublin	MAA
ERR363342	ERR363342	EAI	UK	MAA
ERR014*	ERR5384772	EAI	Norway	MAA
ERR459790	ERR459790	EAI	Australia	MAA
ERR337811	ERR337811	EAI	UK	MAA
ERR369277	ERR369277	EAI	UK	MAA
ERR363459	ERR363459	EAI	DENMARK	MAA
ERR459853	ERR459853	EAI	Australia	MAA
SRR3321818	SRR3321818	EAI	Michigan	MAA
ERR373976	ERR373976	EAI	US	MAA
ERR459773	ERR459773	EAI	Australia	MAA
ERR001*	ERR5384759	EAI	Norway	MAA
ERR373990	ERR373990	EAI	US	MAA
ERR459856	ERR459856	EAI	Australia	MAA
ERR373991	ERR373991	EAI	US	MAA
ERR459921	ERR459921	EAI	Australia	MAA
SRR901527	SRR901527	EAI	Maryland	MAA
ERR363256	ERR363256	EAI	UK	MAA
ERR459959	ERR459959	EAI	Australia	MAA
ERR373954	ERR373954	EAI	US	MAA
ERR369329	ERR369329	EAI	UK	MAA
ERR484983	ERR484983	EAI	Australia	MAA
ERR115035	ERR115035	DCC1	UK	MAA
ERR340533	ERR340533	DCC1	UK	MAA
ERR485004	ERR485004	DCC1	UK	MAA
ERR340547	ERR340547	DCC1	UK	MAA
ERR340553	ERR340553	DCC1	UK	MAA
ERR340546	ERR340546	DCC1	UK	MAA
ERR115026	ERR115026	DCC1	UK	MAA
ERR363258	ERR363258	DCC1	UK	MAA
ERR340563	ERR340563	DCC1	UK	MAA
ERR374019	ERR374019	DCC1	US	MAA
ERR374034	ERR374034	DCC1	US	MAA
ERR363247	ERR363247	DCC1	UK	MAA
ERR340568	ERR340568	DCC1	UK	MAA
ERR494932	ERR494932	DCC1	UK	MAA
ERR374010	ERR374010	DCC1	US	MAA
ERR494925	ERR494925	DCC1	UK	MAA
ERR373971	ERR373971	DCC1	US	MAA
ERR340497	ERR340497	DCC1	UK	MAA
ERR374013	ERR374013	DCC1	US	MAA
ERR343230	ERR343230	DCC1	UK	MAA
ERR343238	ERR343238	DCC1	UK	MAA
ERR340520	ERR340520	DCC1	UK	MAA
ERR369263	ERR369263	DCC1	UK	MAA
ERR363324	ERR363324	DCC1	Dublin	MAA

ERR115039	ERR115039	DCC1	UK	MAA
ERR373948	ERR373948	DCC1	US	MAA
ERR484953	ERR484953	DCC1	UK	MAA
ERR340565	ERR340565	DCC1	UK	MAA
ERR363330	ERR363330	DCC1	UK	MAA
ERR369153	ERR369153	DCC1	UK	MAA
ERR369141	ERR369141	DCC1	DENMARK	MAA
ERR369221	ERR369221	DCC1	UK	MAA
ERR494973	ERR494973	DCC1	UK	MAA
ERR484954	ERR484954	DCC1	UK	MAA
ERR343184	ERR343184	DCC1	UK	MAA
ERR459814	ERR459814	DCC1	Australia	MAA
ERR485003	ERR485003	DCC1	UK	MAA
ERR115066	ERR115066	DCC1	UK	MAA
ERR369154	ERR369154	DCC1	UK	MAA
ERR115047	ERR115047	DCC1	UK	MAA
ERR340536	ERR340536	EAI	UK	MAA
ERR484948	ERR484948	EAI	UK	MAA
ERR363254	ERR363254	DCC2	UK	MAA
SRR3321824	SRR3321824	DCC2	Michigan	MAA
ERR459830	ERR459830	DCC2	Australia	MAA
ERR363244	ERR363244	DCC2	UK	MAA
ERR459966	ERR459966	DCC2	Australia	MAA
ERR349280	ERR349280	DCC2	US	MAA
ERR340569	ERR340569	DCC2	UK	MAA
ERR490691	ERR490691	DCC2	UK	MAA
ERR459792	ERR459792	DCC2	Australia	MAA
ERR019*	ERR5384777	DCC2	Norway	MAA
ERR369146	ERR369146	DCC2	DENMARK	MAA
ERR459970	ERR459970	DCC2	Dublin	MAA
ERR374020	ERR374020	DCC2	US	MAA
ERR369167	ERR369167	DCC2	US	MAA
ERR340522	ERR340522	DCC2	UK	MAA
ERR494947	ERR494947	DCC2	Australia	MAA
SRR343084	SRR343084	DCC2	Maryland	MAA
ERR459974	ERR459974	DCC2	Dublin	MAA
ERR363433	ERR363433	DCC2	US	MAA
ERR484996	ERR484996	DCC2	UK	MAA
ERR363339	ERR363339	DCC2	UK	MAA
ERR484955	ERR484955	DCC2	UK	MAA
ERR340572	ERR340572	DCC2	UK	MAA
ERR349276	ERR349276	DCC2	US	MAA
ERR494936	ERR494936	DCC2	UK	MAA
ERR373983	ERR373983	DCC2	US	MAA
ERR363411	ERR363411	DCC2	US	MAA
ERR373972	ERR373972	DCC2	US	MAA
ERR340530	ERR340530	DCC2	UK	MAA
ERR459939	ERR459939	DCC2	Australia	MAA
ERR373951	ERR373951	DCC2	US	MAA
ERR459788	ERR459788	DCC2	Australia	MAA
ERR494886	ERR494886	DCC2	UK	MAA
ERR494835	ERR494835	DCC2	UK	MAA
ERR373975	ERR373975	DCC2	US	MAA

ERR373989	ERR373989	DCC2	US	MAA
ERR459822	ERR459822	DCC2	Australia	MAA
ERR459922	ERR459922	DCC2	Australia	MAA
ERR373977	ERR373977	DCC2	US	MAA
SRR3321823	SRR3321823	DCC2	Michigan	MAA
ERR363323	ERR363323	EAI	Dublin	MAA
ERR340496	ERR340496	EAI	UK	MAA
ERR002*	ERR5384760	EAI	Norway	MAA
ERR369209	ERR369209	EAI	UK	MAA
ERR363269	ERR363269	EAI	UK	MAA
ERR374012	ERR374012	EAI	US	MAA
ERR459865	ERR459865	EAI	Dublin	MAA
ERR363255	ERR363255	EAI	UK	MAA
ERR494941	ERR494941	EAI	UK	MAA
ERR363267	ERR363267	EAI	UK	MAA
ERR363321	ERR363321	EAI	Dublin	MAA
ERR363261	ERR363261	EAI	UK	MAA
ERR020*	ERR5384760	EAI	Norway	MAA
ERR369196	ERR369196	EAI	UK	MAA
ERR343220	ERR343220	EAI	UK	MAA
ERR363360	ERR363360	EAI	UK	MAM
ERR374029	ERR374029	EAI	US	MAM
ERR340518	ERR340518	EAI	UK	MAM
ERR363242	ERR363242	EAI	UK	MAM
ERR369294	ERR369294	EAI	UK	MAM
ERR340564	ERR340564	EAI	UK	MAM
ERR373985	ERR373985	EAI	US	MAM
ERR374030	ERR374030	EAI	US	MAM
ERR046*	ERR5384815	EAI	Norway	MAM
ERR048*	ERR5384817	EAI	Norway	MAM
ERR484993	ERR484993	EAI	UK	MAM
ERR340537	ERR340537	EAI	UK	MAM
ERR459926	ERR459926	EAI	Australia	MAM
ERR459832	ERR459832	EAI	Australia	MAM
ERR373942	ERR373942	EAI	DENMARK	MAM
ERR459940	ERR459940	EAI	Australia	MAM
ERR459802	ERR459802	EAI	Australia	MAM
ERR052*	ERR5384821	EAI	Norway	MAM
ERR369309	ERR369309	EAI	UK	MAM
ERR494907	ERR494907	DCC	UK	MAM
ERR374007	ERR374007	DCC	US	MAM
ERR351973	ERR351973	DCC	UK	MAM
ERR340548	ERR340548	DCC	UK	MAM
ERR340517	ERR340517	DCC	UK	MAM
ERR459810	ERR459810	DCC	Australia	MAM
ERR459972	ERR459972	DCC	Dublin	MAM
ERR036*	ERR5384805	DCC	Norway	MAM
ERR369144	ERR369144	DCC	DENMARK	MAM
ERR349272	ERR349272	DCC	US	MAM
ERR369198	ERR369198	DCC	UK	MAM
ERR115074	ERR115074	DCC	UK	MAM
ERR484963	ERR484963	DCC	UK	MAM
ERR047*	ERR5384816	DCC	Norway	MAM

ERR343180	ERR343180	DCC	UK	MAM
ERR034*	ERR5384803	DCC	Norway	MAM
ERR343194	ERR343194	DCC	UK	MAM
SRR315360	SRR315360	DCC	Maryland	MAM
SRR341226	SRR341226	DCC	Maryland	MAM
SRR315358	SRR315358	DCC	Maryland	MAM
SRR340137	SRR340137	DCC	Maryland	MAM
ERR369292	ERR369292	DCC	UK	MAM
ERR369275	ERR369275	DCC	UK	MAM
ERR041*	ERR5384810	DCC	Norway	MAM
ERR459858	ERR459858	DCC	Australia	MAM
ERR459771	ERR459771	DCC	Australia	MAM
ERR459927	ERR459927	DCC	Australia	MAM
ERR490687	ERR490687	DCC	UK	MAM
ERR369300	ERR369300	DCC	UK	MAM
ERR494837	ERR494837	DCC	UK	MAM
SRR3321817	SRR3321817	DCC	Michigan	MAM
ERR369178	ERR369178	DCC	UK	MAM
ERR369289	ERR369289	DCC	UK	MAM
ERR369308	ERR369308	DCC	UK	MAM
ERR369326	ERR369326	DCC	UK	MAM
SRR3321822	SRR3321822	DCC	Michigan	MAM
SRR3321826	SRR3321826	DCC	Michigan	MAM
ERR369170	ERR369170	DCC	UK	MAM
ERR484990	ERR484990	DCC	UK	MAM
ERR115075	ERR115075	DCC	UK	MAM
SRR315266	SRR315266	EAI	Maryland	MAM
ERR369155	ERR369155	EAI	UK	MAM
ERR050*	ERR5384819	EAI	Norway	MAM
ERR484957	ERR484957	EAI	UK	MAM
ERR369197	ERR369197	EAI	UK	MAM
ERR494928	ERR494928	EAI	UK	MAM
ERR340558	ERR340558	EAI	UK	MAM
ERR340540	ERR340540	EAI	UK	MAM
ERR051*	ERR5384820	EAI	Norway	MAM
ERR373970	ERR373970	EAI	US	MAM
SRR3321827	SRR3321827	EAI	Michigan	MAM
ERR340544	ERR340544	EAI	UK	MAM
SRR315577	SRR315577	EAI	Maryland	MAM
ERR369246	ERR369246	EAI	UK	MAM
SRR3666120	SRR3666120	EAI	Michigan	MAM
ERR363442	ERR363442	EAI	DENMARK	MAM
ERR373979	ERR373979	EAI	US	MAM
ERR459847	ERR459847	EAI	HOLLAND	MAM
ERR490670	ERR490670	EAI	UK	MAM
ERR363422	ERR363422	EAI	DENMARK	MAM
SRR3321819	SRR3321819	EAI	Michigan	MAM

Table S2. Pangenome calculations based on 3 different datasets: both subspecies together and separate. Homologous gene families determined using 95% amino acid similarity.

Dataset	Num Isolates	Gene Group	Gene Freq.	Num Genes
MAA & MAM	200	Core	99-100%	3672
		Cloud	0-15%	39518
		Total	0-100%	45611
MAA	120	Core	99-100%	3877
		Cloud	0-15%	25419
		Total	0-100%	31141
MAM	80	Core	99-100%	3653
		Cloud	0-15%	20397
		Total	0-100%	26061

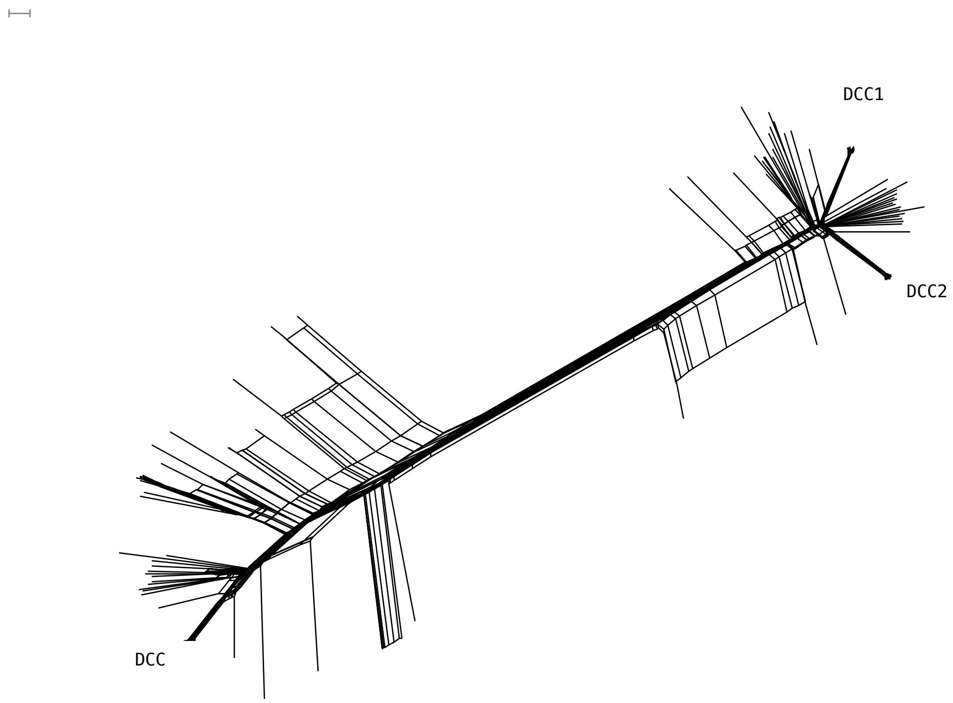

Figure S1. Core genome phylogenetic network contains long branch delineating subspecies. Core genome phylogenetic network of *M. abscessus* subspecies *abscessus* (MAA, right) and subspecies *massiliense* (MAM, left). Reticulations indicate uncertainty in placement of that isolate (tip) in the network, which can be the result of recombination. Subspecies MAA and MAM are separated by a long branch. DCCs are found in both subspecies with few reticulations.

A

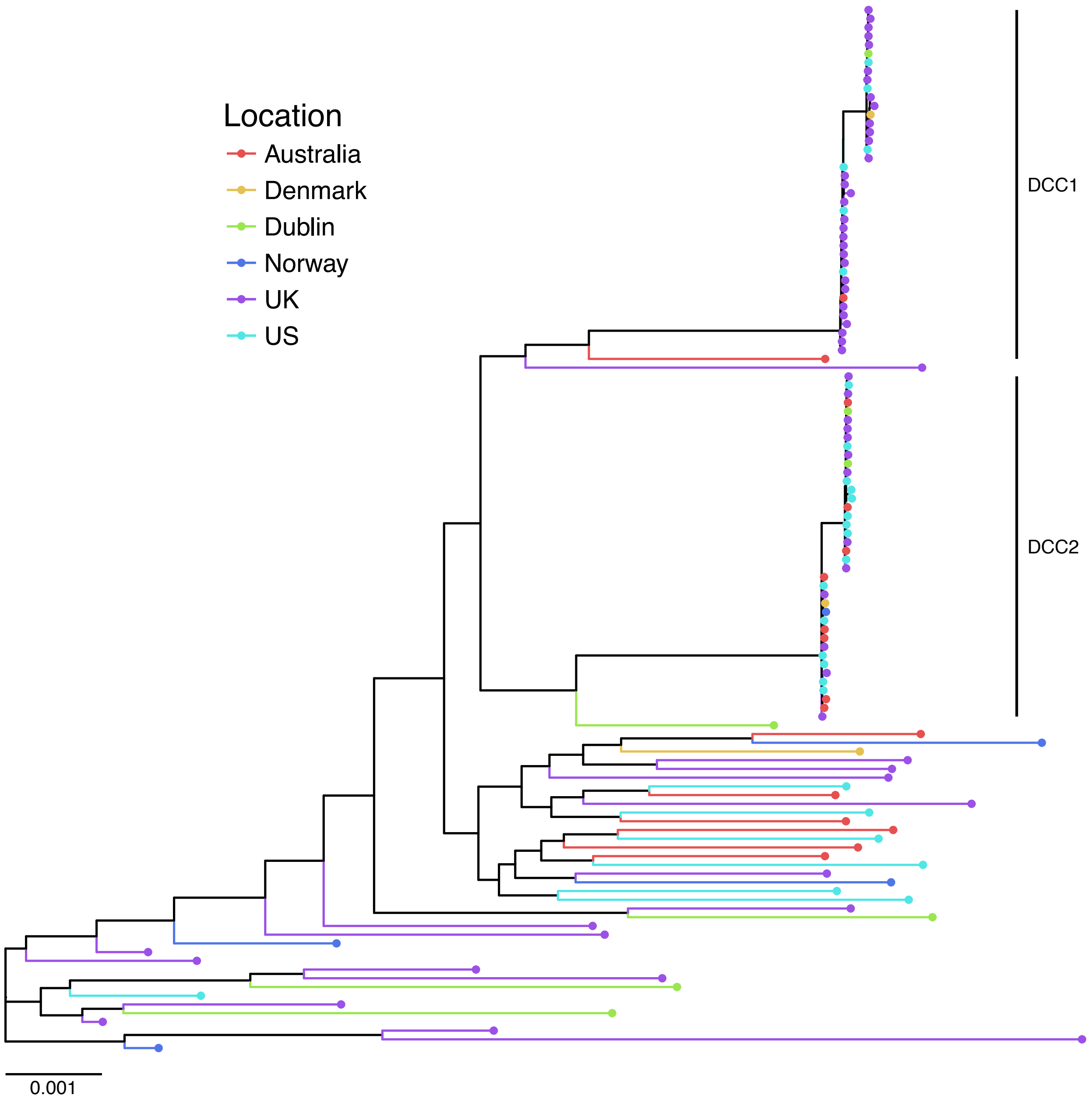
Location
Australia
Denmark
Dublin
Norway
UK
US
DCC1
DCC2
0.001

B

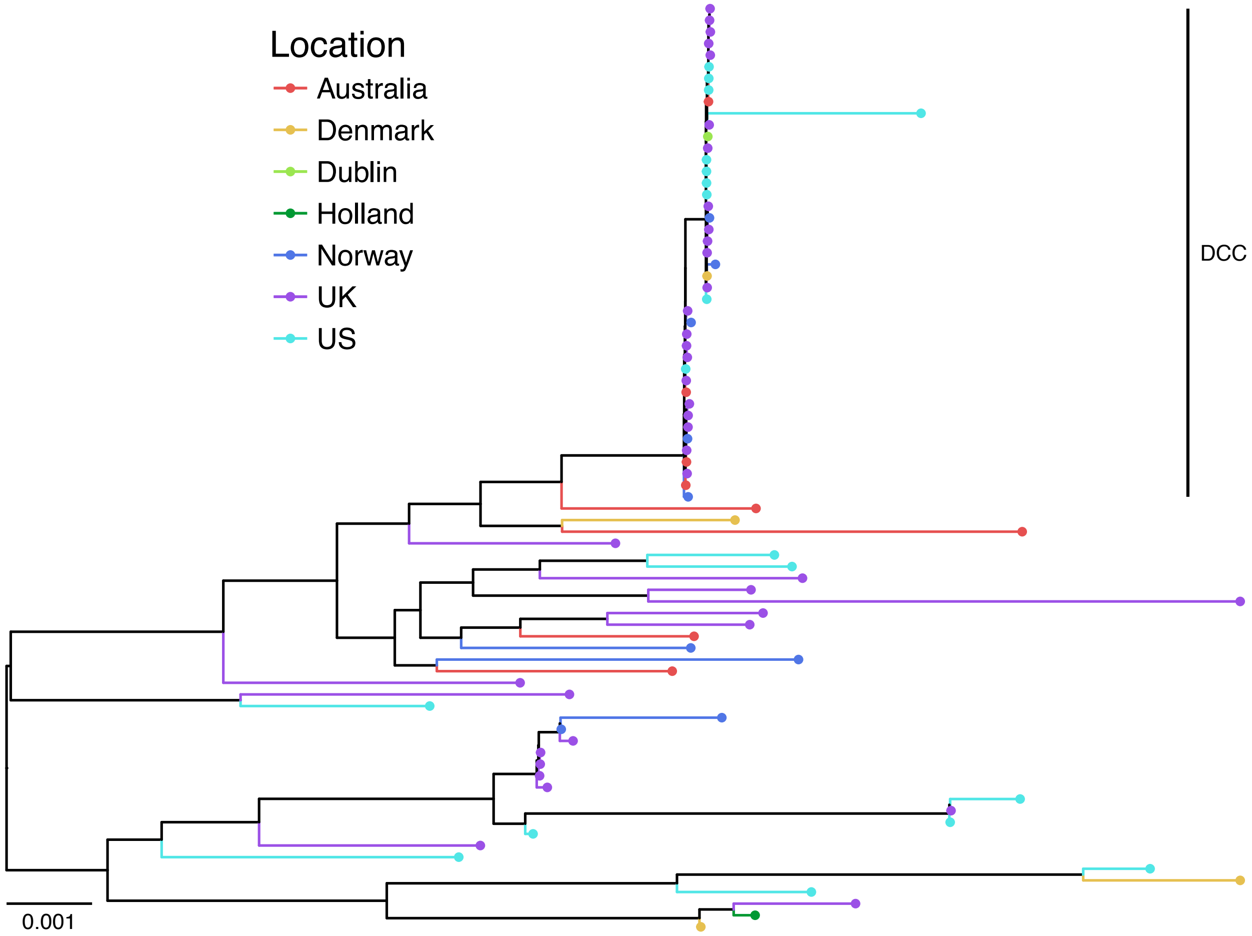

Figure S2. Maximum likelihood phylogenies of the core genomes of *M. abscessus* subspecies show distinct structure of Dominant Circulating Clones (DCC) and Environmentally Acquired Isolates (EAI). We inferred a maximum likelihood phylogeny from core genome alignments of (A) 120 MAA isolates and (B) 80 MAM isolates. DCCs are labeled in both subspecies according to Bryant *et al.* 2016. Non-DCC isolates are referred to as EAIs. There is little geographic structure in DCCs from either subspecies, consistent with their global transmission. The phylogeny is midpoint rooted, and branch lengths are scaled by the number of substitutions per site.

A

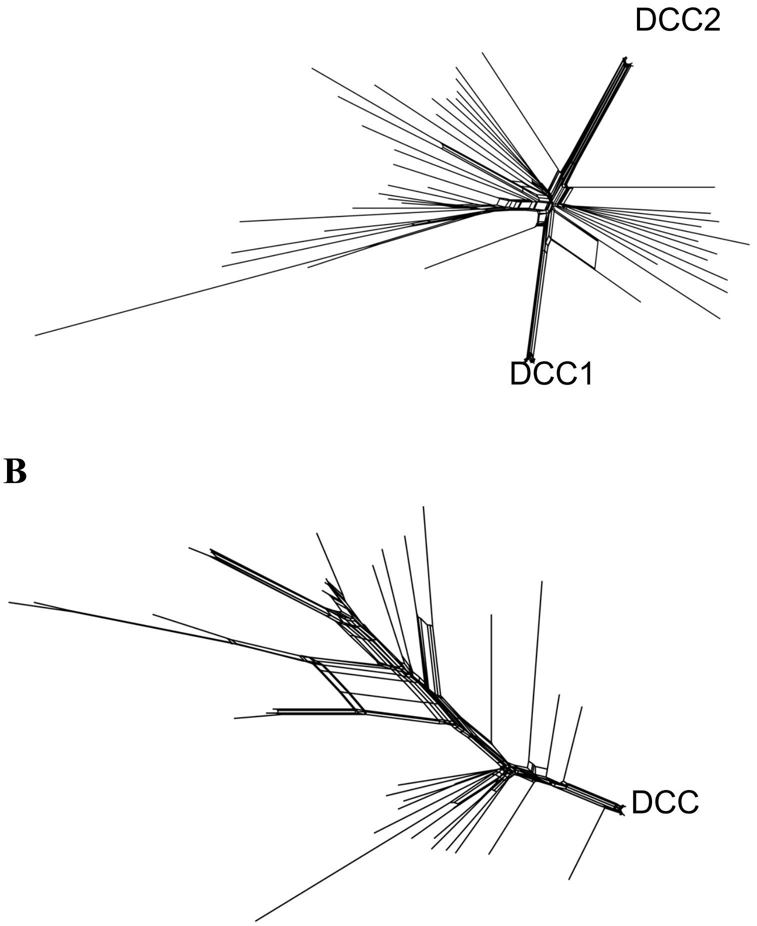

Figure S3. Core genome networks of both *M. abscessus* subspecies contain reticulations and distinct branches to the DCCs. Core genome phylogenetic network of *M. abscessus* (A) subspecies *abscessus* (MAA) and (B) subspecies *massiliense* (MAM). Reticulations indicate uncertainty in placement of that isolate (tip) in the network, which can be the result of recombination. DCCs are found in both subspecies with few reticulations and distinct branching. MAM appears to contain more reticulations than MAA, suggesting MAM participates in LGT more frequently. The core genomes of both subspecies contain evidence of recombination (PHI test for recombination, p=0.0).

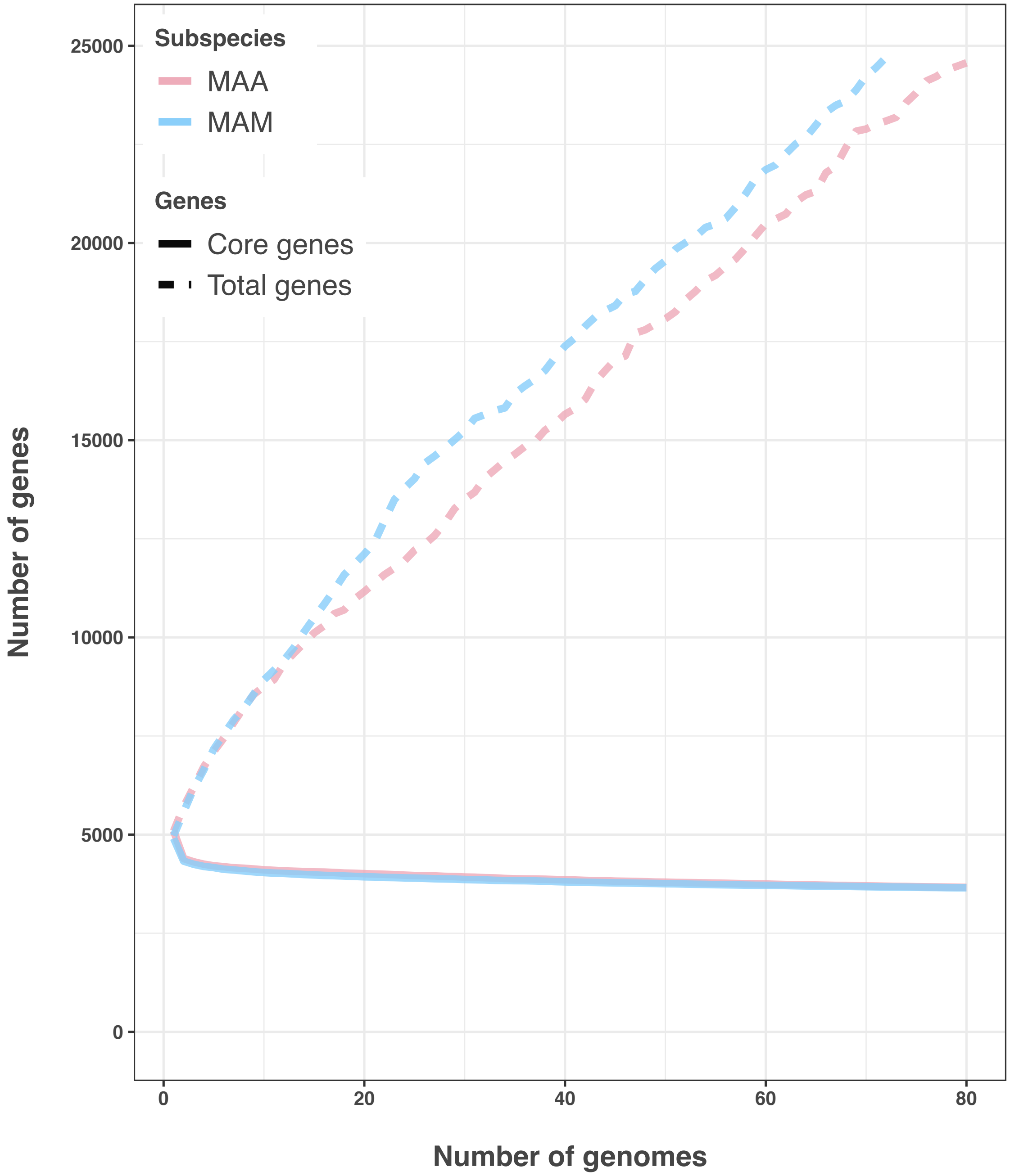

Figure S4. ***M. abscessus*** **subspecies pangenome sizes are similar.** Rarefaction and accumulation curves of core and total gene content for MAA and MAM. MAA isolates were subsampled to the number of MAM isolates, and both subspecies were iteratively sampled 100 times. The median value is shown. The number of genes in the core and pangenomes is similar in both subspecies MAA and MAM.

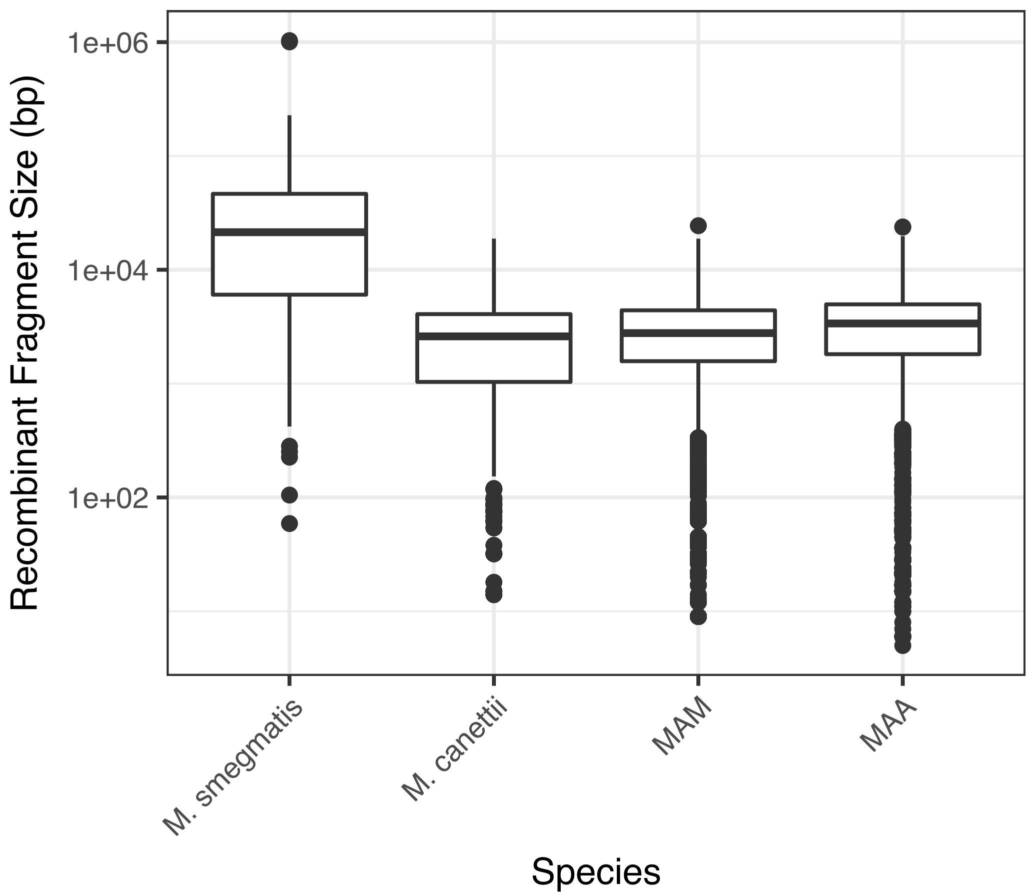

Figure S5. Recombinant fragment length distribution. Boxplot of recombinant fragment lengths in *M. smegmatis, M. canettii,* MAM, and MAM. LGT fragment lengths for *M. smegmatis* and *M. canettii* were previously published data (Mortimer and Pepperell, 2014). The box spans the interquartile range, the median is represented by the middle line, and the whiskers extend to +/- 1.5 times the interquartile range. Data beyond the whiskers are outlying points and plotted individually. The distributions of recombinant fragment lengths for MAA and MAM is comparable to that of *M. canettii*, a species known to participate in Distributive Conjugal Transfer (DCT). This suggests DCT may be an important mechanism of LGT for *M. abscessus*.

A

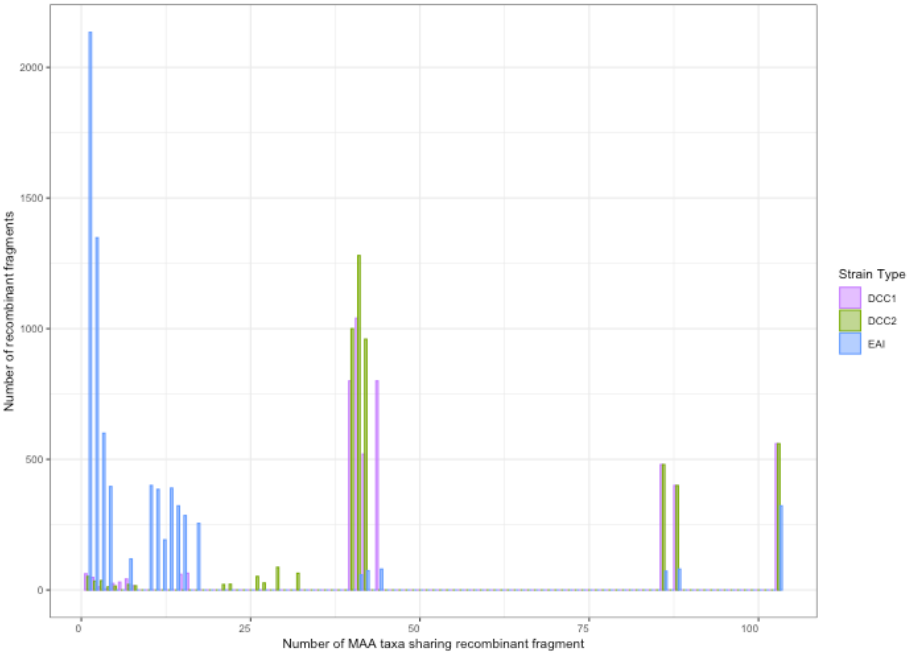

B

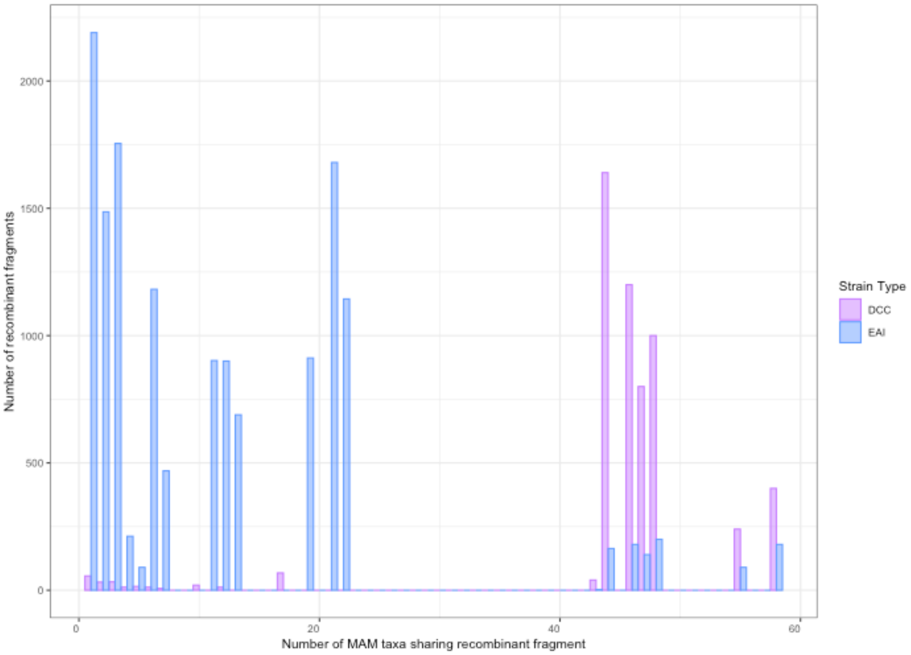

Figure S6. Environmentally Acquired Isolates (EAIs) are more likely to have unique recombinant fragments than Dominant Circulating Clones (DCCs). Histograms of the recombinant fragment counts in subspecies MAA (A) and subspecies MAM (B) that are shared across one or more isolates. In both subspecies, DCCs are more likely to have fragments shared across the entire clade, whereas EAIs are more likely to have recombinant fragments unique to a single or a few individuals. This suggests that LGT is more frequent in EAIs than in DCCs and/or the DCCs are more recently diverged such that recombinant fragments have been vertically transmitted and have not yet been interrupted by recurrent recombination events.

A

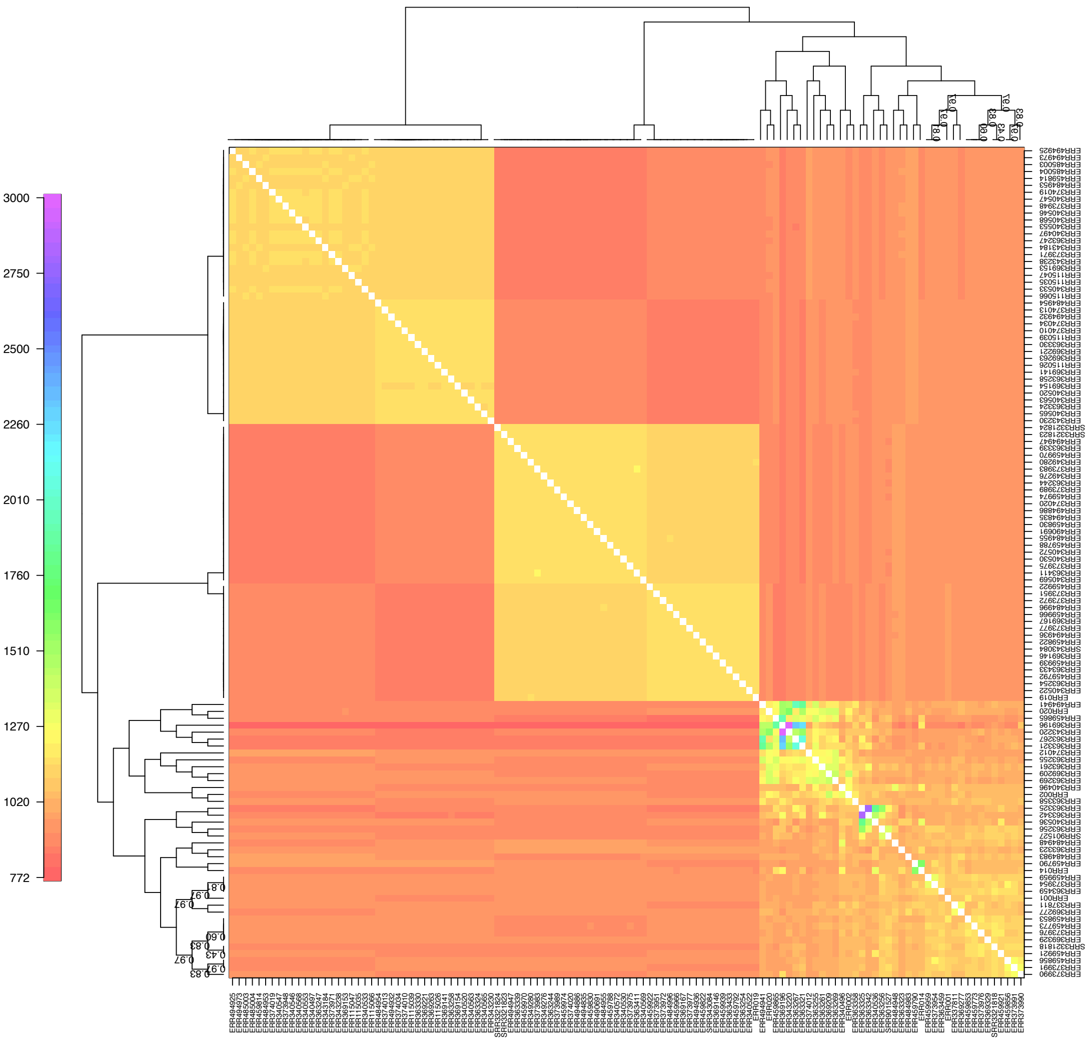

B

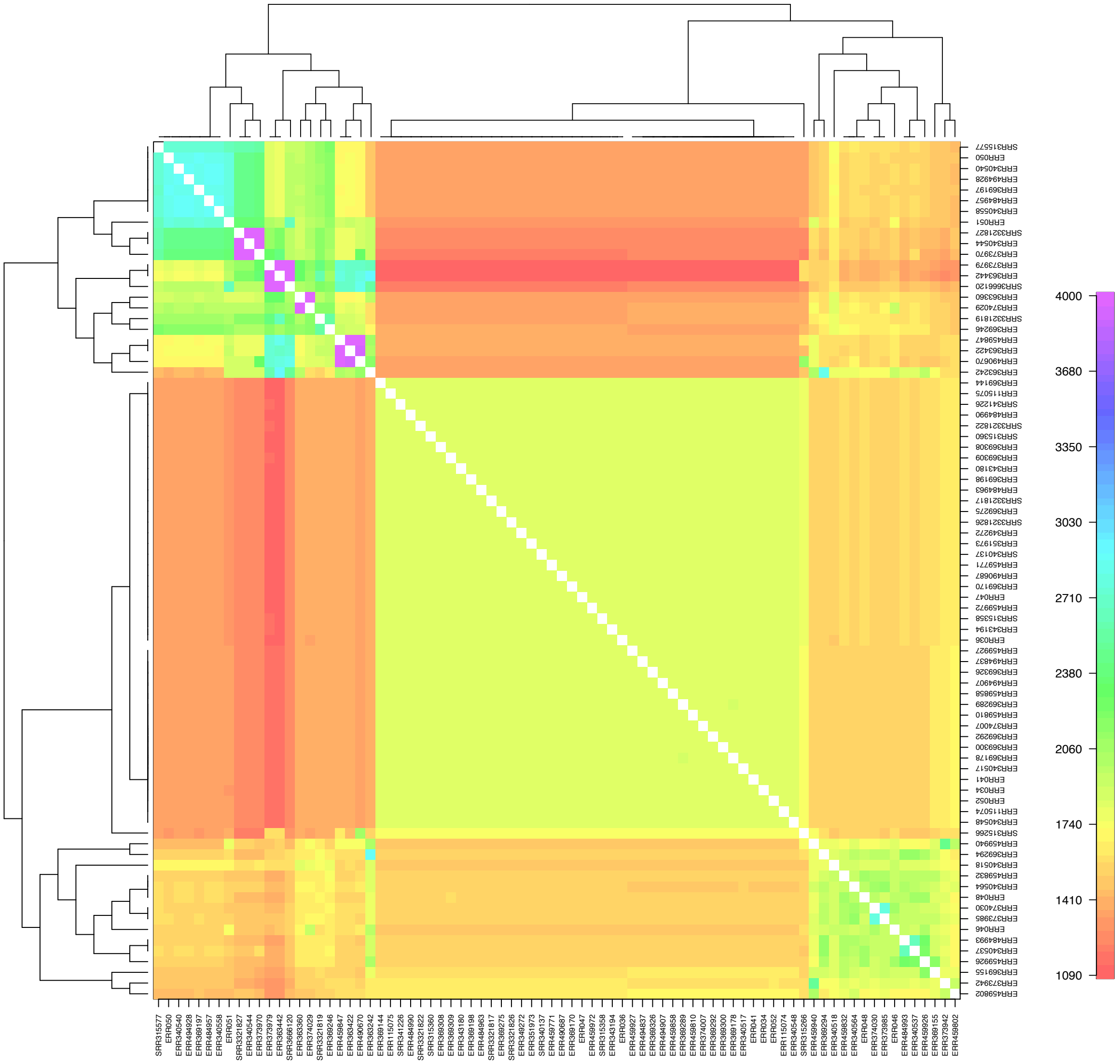

Figure S7. Co-ancestry matrices depict population structure and gene flow in *M. abscessus* subspecies' core genomes. Co-ancestry matrices based on the core genomes of (A) subspecies MAA and (B) subspecies MAM. Co-ancestry matrices are colored by the estimated fractions of chunks in the genome that are imported from a donor genome (column) to a recipient genome (row). For example, colors higher on the relative scale (right) indicate a higher proportion of transferred fragments. The trees on the top and left of each matrix show clustering assignments as determined by FineSTRUCTURE. Both subspecies depict strong population structure of the Dominant Circulating Clones (DCCs), as shown by the two orange squares in MAA (A) and the large lime green square in the middle of the MAM matrix (B). Additionally, in both subspecies, the Environmentally Acquired Isolates (EAIs) appear to have more recombination relative to the DCCs.

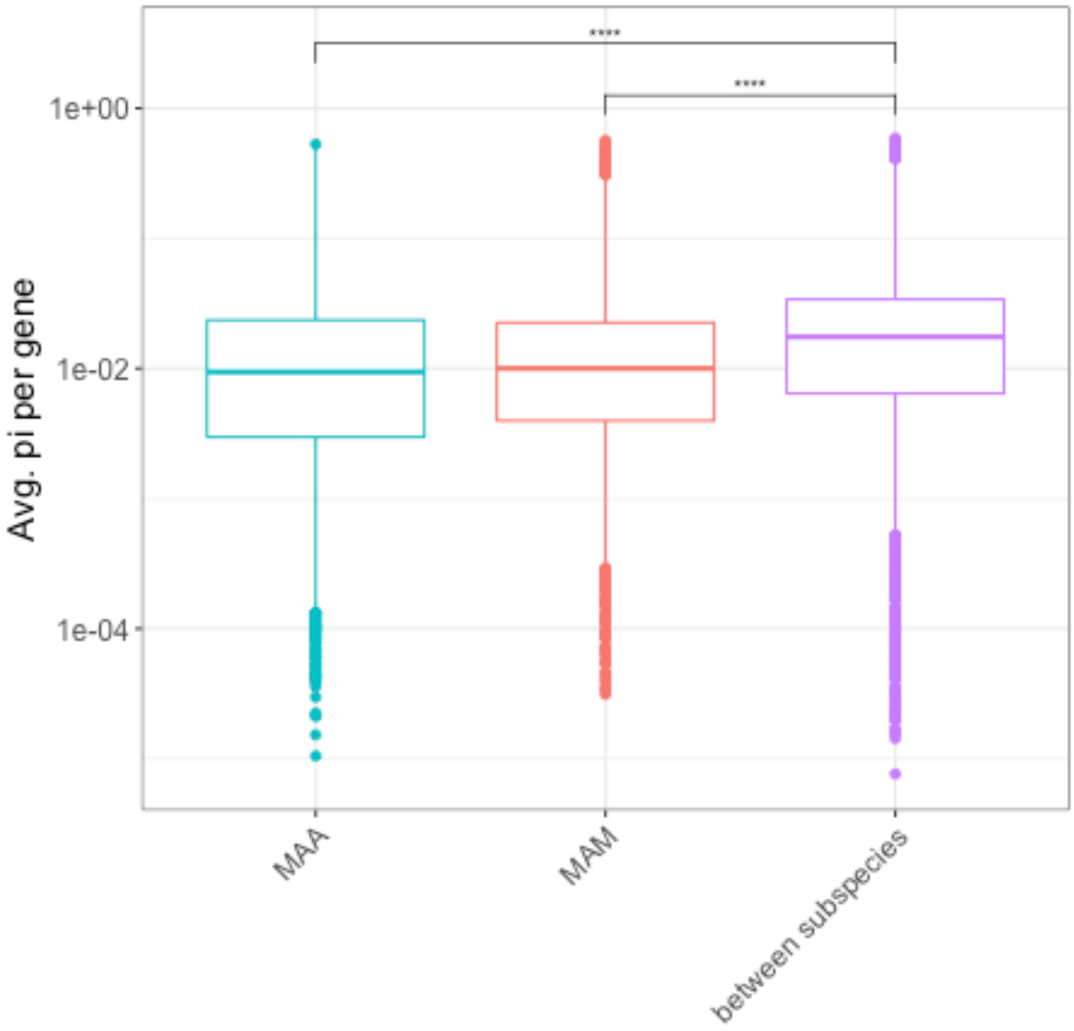

Figure S8. Shared accessory genes are genetically differentiated between subspecies. The distributions of nucleotide diversity (π) per shared accessory gene within and between subspecies were calculated and log transformed. The box spans the interquartile range, the median is represented by the middle line, and the whiskers extend to +/- 1.5 times the interquartile range. Data beyond the end of the whiskers are outlying points and plotted individually. In the shared accessory genome, average gene π values differ significantly by group (Kruskal-Wallis test, H = 2786, $p < 0.0001$). The distributions of average gene π values within subsp. MAA (Mann-Whitney-Wilcoxon, W = 1.4e7, $p = 3.02 \times 10^{-12}$) and subsp. MAM (Mann-Whitney-Wilcoxon, W = 3.4e7, $p = 4.4 \times 10^{-16}$) are lower than in comparisons between subspecies. This is consistent with accessory gene flow occurring more often within subspecies than between them. P-value legend: ****: <0.0001.

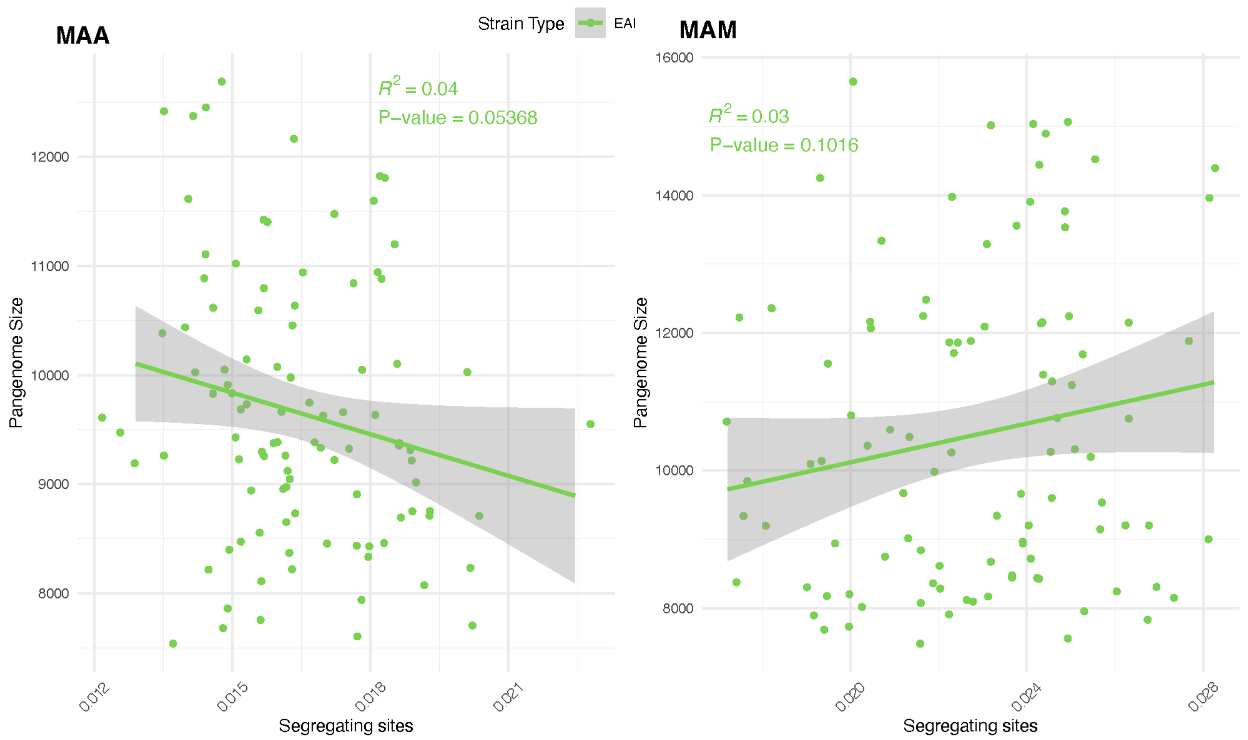

Figure S9. Pangenome size is not significantly correlated with core genome divergence. We randomly sampled the EAIs, measured segregating sites as a proxy for divergence times and calculated the core and pan genome sizes. Pan genome size was not significantly correlated with sample divergence ($p > 0.05$ for both MAA and MAM).

A

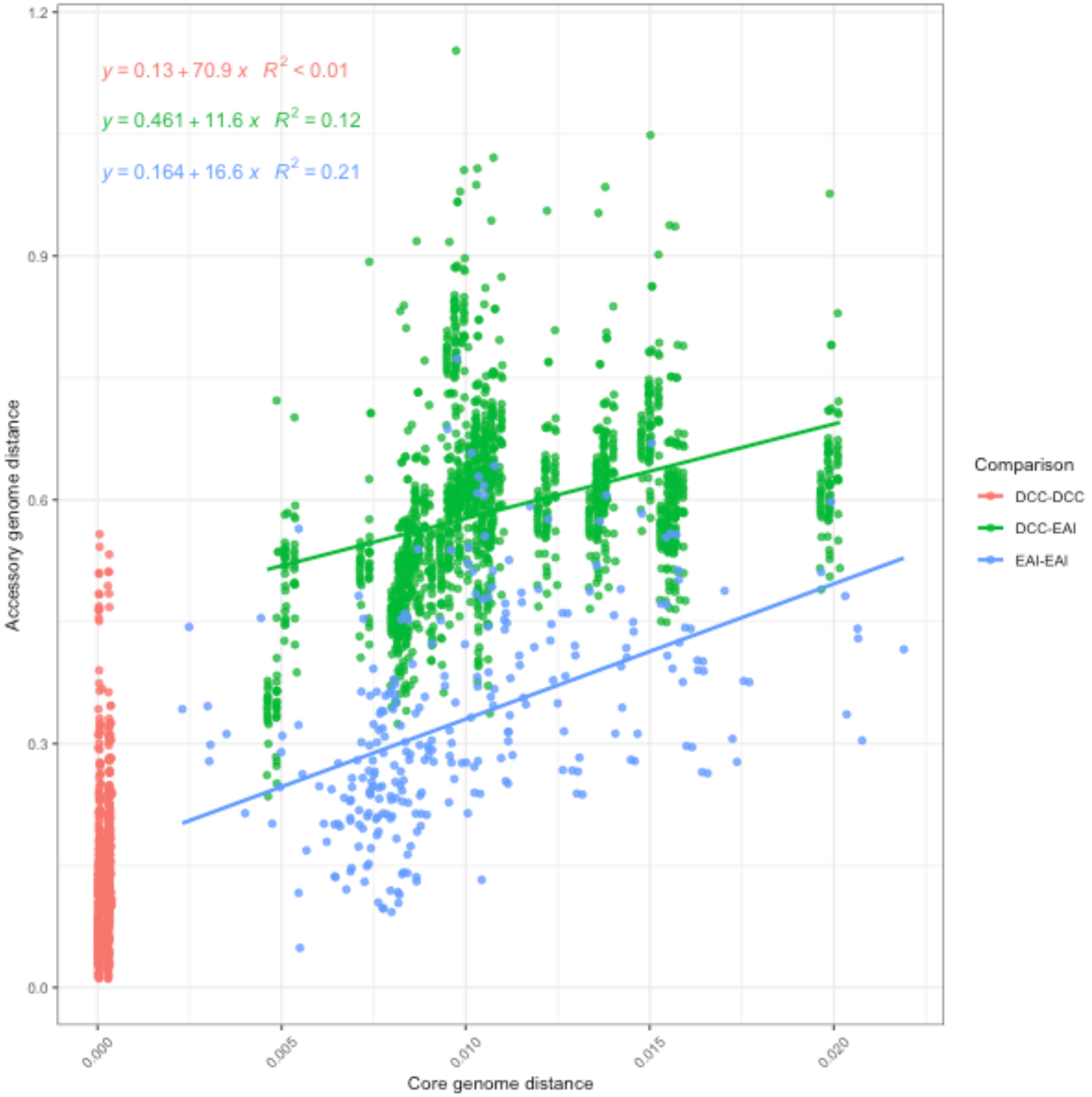

y = 0.13 + 70.9 x R^2 < 0.01
y = 0.461 + 11.6 x R^2 = 0.12
y = 0.164 + 16.6 x R^2 = 0.21
1.2
0.9
0.6
0.3
0.0
Accessory genome distance
0.000
0.005
0.010
0.015
0.020
Core genome distance
Comparison
DCC-DCC
DCC-EAI
EAI-EAI

B

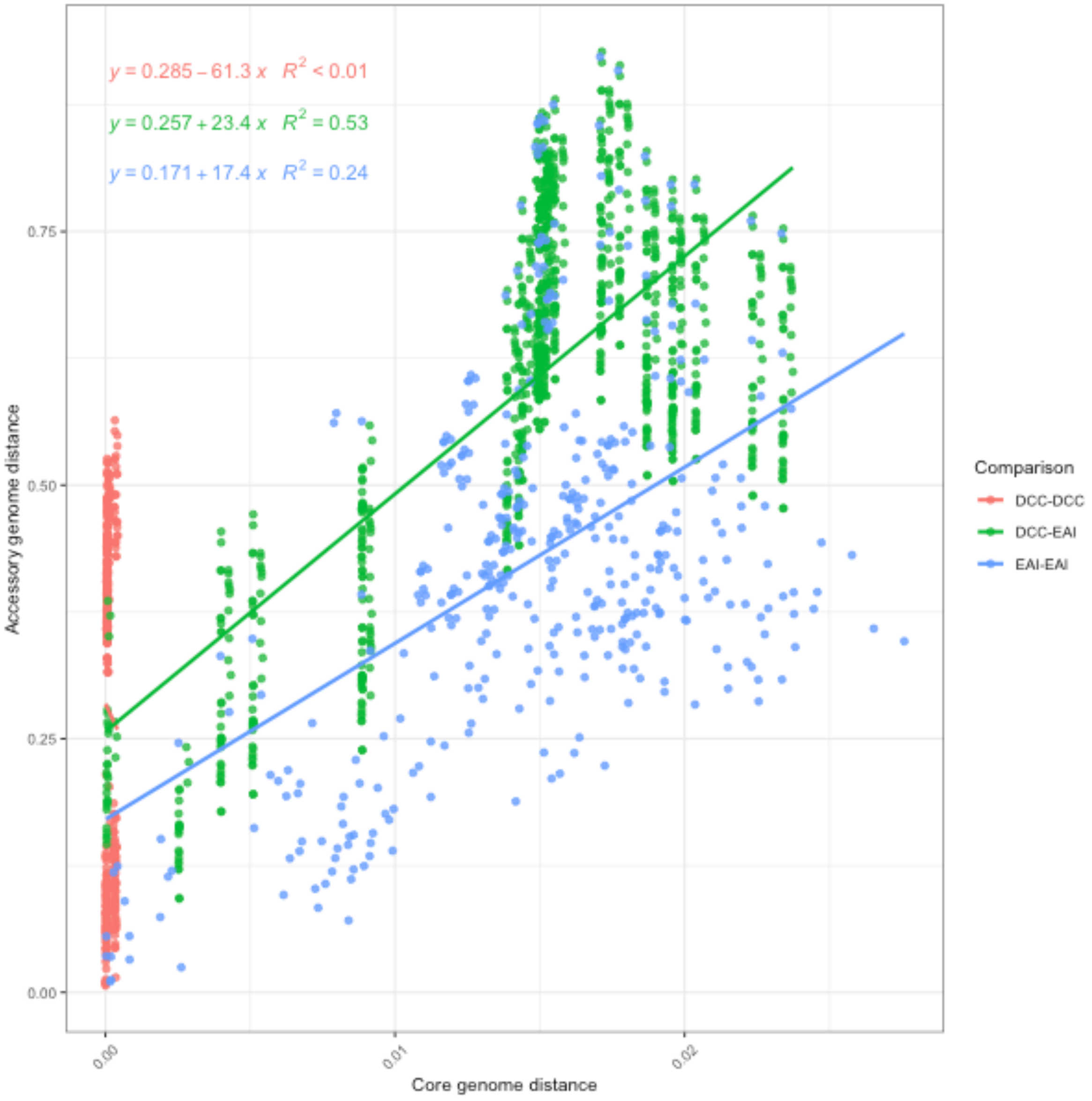

Figure S10. DCCs appear genetically isolated from EAIs. Accessory genome distance was calculated as the distance between tips of a phylogenetic tree inferred from a presence/absence matrix of accessory genes identified in each isolate (Y axis). This was plotted against core genome distance as measured by the distance between tips of the core genome phylogeny (X axis). The pace of accessory genome differentiation is greater for MAM (panel B) than for MAA (panel A), reflected in higher slopes of regressions. For a given core genome distance, DCCs are more differentiated from EAIs than EAIs are from each other. This is consistent with cessation of gene flow from EAIs to DCCs and overall reduction of LGT among DCCs.

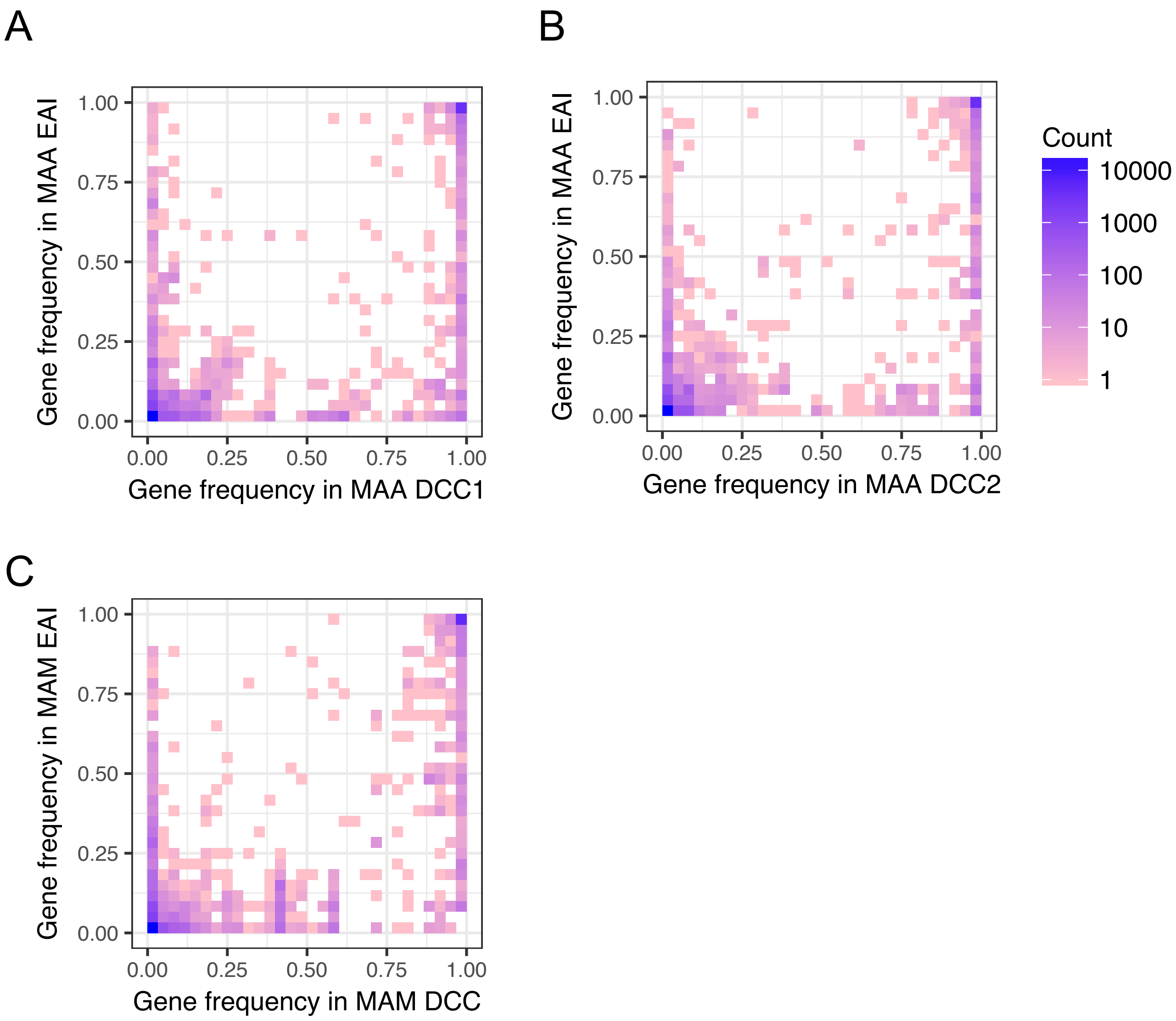

Figure S11. Gene content variation in DCCs with respect to EAIs. Heat map of pangenome gene frequencies compared across DCCs and EAIs in subspecies MAA (A, B) and MAM (C). Much of the accessory gene content is rare, and genes are not maintained at similar frequencies. A: MAA DCC1 shows evidence of remodeling of its core genome relative to EAIs, with both loss of what was previously core gene content (upper left corner) and gain of novel core gene content (lower right corner). B: MAA DCC2 shows evidence of gain only (upper left quadrant is blank, lower right is filled). C: MAM DCC exhibits neither loss nor gain (upper left and lower right corners are blank). This suggests the DCCs may have emerged successively, with MAA DCC1 the most mature pathogenic emergence.

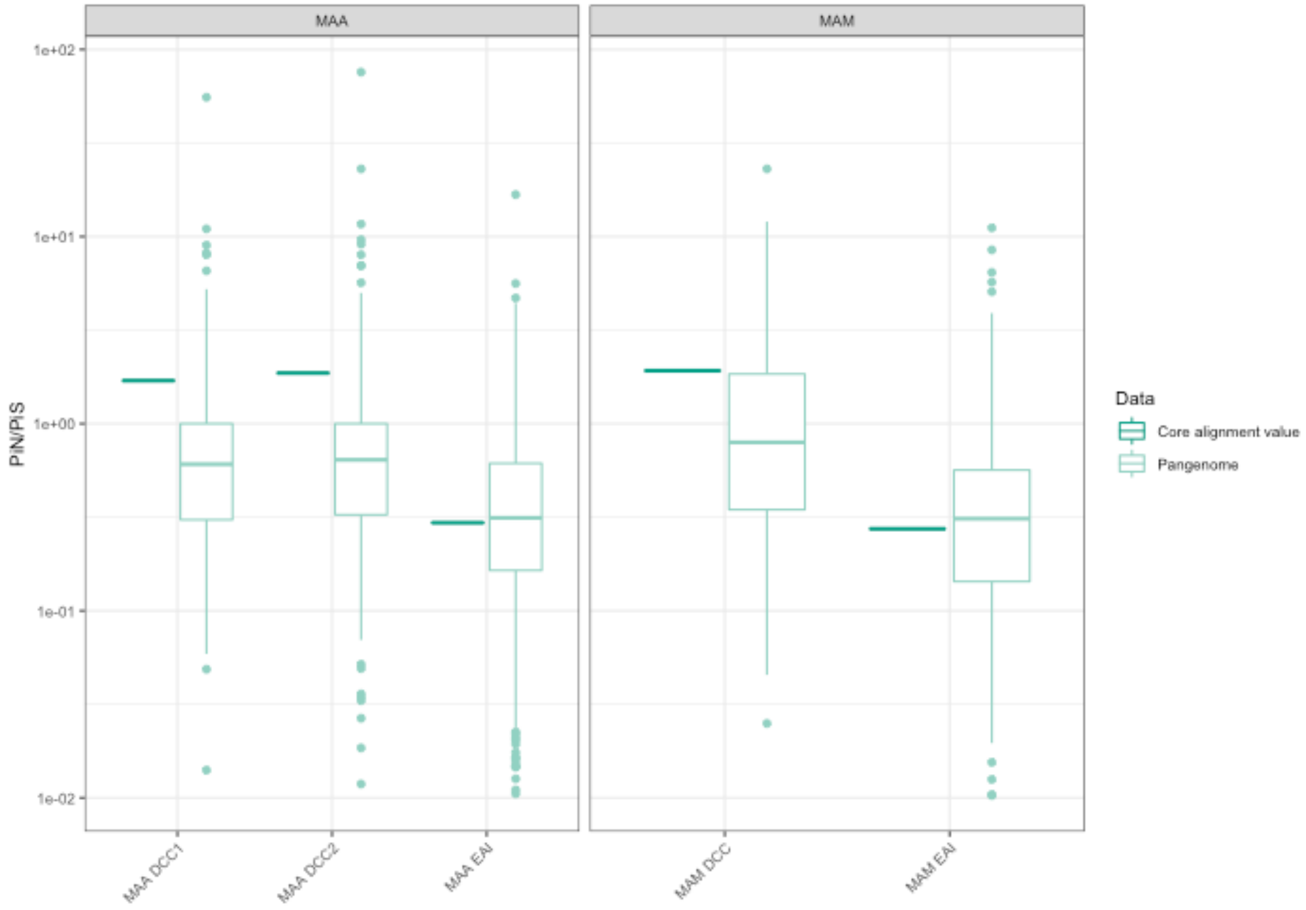

Figure S12. Purifying selection is relaxed in the pangenomes of the DCCs. Distributions of average $\pi N/\pi S$ per gene across the pangenomes of subsp. MAA and subsp. MAM. Core and pangenome values of $\pi N/\pi S$ are higher in DCCs than in EAIs, a difference that is statistically significant (MAA DCC1 vs EAI: Mann-Whitney-Wilcoxon test, $W = 2.9e5$, $p = 0.0003$): the DCCs appear to be under relaxed purifying selection relative to the EAIs within each *M. abscessus* subspecies. Boxes in plots span the interquartile range, the median is represented by the middle line, and the whiskers extend to +/- 1.5 times the interquartile range. Data beyond the whiskers are outlying points and plotted individually.
